# Investigating degradation mechanisms in organic light-emitting diodes using operando electrically pumped spectroscopy

**DOI:** 10.1038/s41377-026-02373-8

**Published:** 2026-07-06

**Authors:** Chang Min Lee, Hyun Jae Lee, Insung Ha, Mengdi Fu, Muhammad Waheed, Geon Lee, Deepak Rajaram Patil, Justin Jesuraj P, Jae Woo Lee, Chul Hoon Kim, Seung Yoon Ryu

**Affiliations:** 1 Department of Physics, Dongguk University, Seoul, 04620 Republic of Korea; 2 Department of Physics and Nanotechnology, SRM institute of Science and Technology-Kattankulathur, Chengalpattu, 6032033 India; 3 Centre of Excellence in Materials for Advanced Technologies (CeMAT), SRM institute of Science and Technology-Kattankulathur, Chengalpattu, 6032033 India; 4 Education and Research Center for Artificial intelligence, Smart Convergence Technology and Department of Electronics and Information Engineering, Korea University, Sejong City, 30019 Republic of Korea; 5 Department of Advanced Materials Chemistry, Korea University, Sejong, 30019 Republic of Korea; 6 Division of Smart Energy Convergence Engineering, Korea University, Sejong, 30019 Republic of Korea; 7 Photoenergy Harvesting and Conversion Technology (PHCT), Dongguk University, Seoul, 04620 Republic of Korea

**Keywords:** Optical spectroscopy, Photonic devices, Optoelectronic devices and components, Organic LEDs

## Abstract

Organic light-emitting diodes (OLEDs) have been analyzed using a variety of techniques, each offering unique insights into their performance and degradation, yet conventional electrical diagnostics such as impedance spectroscopy (IS) provide only limited insight into the microscopic origins of failure. This study presents a comprehensive analysis of OLED degradation by integrating IS with operando electrically pumped spectroscopy (EPS), which enables simultaneous electrical and optical probing under operational bias. OLED devices, including systematically fabricated in-situ and ex-situ samples, were investigated under both pristine and thermally degraded conditions. IS effectively captured electrical trends, but could not pinpoint the specific layers or interfaces responsible for efficiency loss in degraded devices. EPS, combined with single-layer and bilayer film analysis, revealed the dominant role of exciton scattering and energy transfer disruptions within the emissive layer (EML) and at the EML/HTL (hole transport layer) interface. Kinetic modeling and decay-associated spectral analysis further identified ultrafast exciton quenching pathways induced by thermal stress. These findings establish EPS as a powerful diagnostic tool for visualizing exciton dynamics and localizing degradation, thereby contributing to the development of more stable OLED device architectures.

## Abstract (web summary)

An integrated operando EPS-based spectroscopic framework enables layer-resolved visualization of exciton dynamics in OLEDs, providing direct insight into degradation pathways under electrical bias.

## Graphical abstract

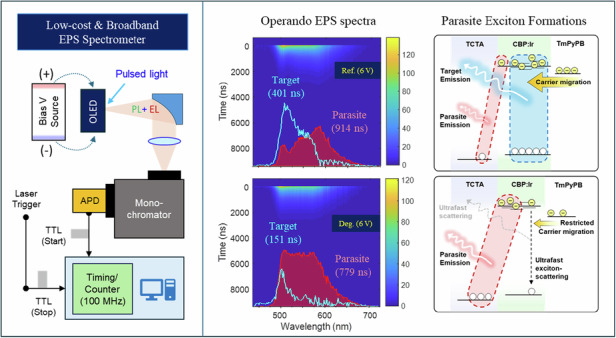

## Introduction

Organic light-emitting diodes (OLEDs) are widely studied for use in diverse applications, from display technologies to biomedical devices. Their simple structure and ability to withstand external stressors make them ideal for next-generation displays. OLEDs are thin, transparent, flexible, offer wide viewing angles, and exhibit high color purity—making them perfect candidates for advanced applications^1–3^. However, several internal challenges must be addressed to enhance their performance and long-term stability, including degradation caused by environmental factors, operational stresses, and fabrication-process problems^4–6^. Factors influencing OLED degradation include exposure to oxygen and moisture, leading to dark spots, oxidation of organic layers, and increased trap densities^7,8^. Heat generated during operation, from processes such as triplet–triplet annihilation (TTA) and triplet–polaron quenching (TPQ), further accelerates degradation through non-radiative recombination and Joule heating^9,10^. Fabrication factors, such as substrate cleanliness and annealing processes, significantly affect device stability and performance^5,11,12^. Various methods have been employed to analyze OLED performance and degradation, gaining insights into their complex dynamics.

Here we employ operando analysis, making real-time observations of device dynamics under actual operating conditions. Although operando techniques have been widely applied in fields such as battery technology and semiconductor materials, their use in optoelectronic devices, including OLEDs, has been relatively limited. Operando techniques have proven valuable in understanding systems like lithium-ion batteries^13,14^, with complex phenomena. For example, since dendrites in batteries grow only during operation, operando analysis is essential for capturing this process^15^. Operando methods, including advanced imaging and spectroscopic techniques such as electrochemical atomic force microscopy (EC-AFM)^16,17^, electrochemical strain microscopy (ESM)^18^, and scanning ion-conductance microscopy (SICM)^19^, have been used to visualize ion dynamics, phase changes, and material degradation in real time, leading to breakthroughs in understanding electrochemical systems at a fundamental level.

Despite the clear benefits of operando methodologies in other material and device fields, their application to optoelectronics, including OLEDs, has remained limited because they rely predominantly on spectrally integrated emission detections^20–25^, which provides no direct information on the emission spectral shape, such as peak position, bandwidth, and asymmetry. To address this gap, we employ broadband electrically pumped spectroscopy (EPS) to investigate degradation mechanisms and detect process abnormalities in green OLEDs, leveraging real-time optical and electrical diagnostics to directly probe internal processes leading to device failure. In this work, we primarily focus on the bias-dependent dynamics of parasite excitons, whose behavior is strongly convoluted by that of the target excitons. Accordingly, broadband EPS measurements with high spectral resolution are essential to disentangle the dynamics of target and parasite excitons in the spectral domain and to quantitatively determine their relative populations in an operating device.

In our previous studies, broadband EPS combined with decay-associated spectra (DAS) analysis was introduced as a feasibility-level approach to examine efficiency roll-off and excitonic interactions in blue OLEDs, including exciplex and electromer formation mediated by triplet–triplet annihilation and triplet–polaron quenching, which influence efficiency and color purity^26^. In general, charge accumulation in organic semiconductors may lead to the formation of polarons and can influence device stability^27,28^. In such cases, exciton–polaron interactions can dissipate exciton energy through non-radiative quenching processes^29^. However, spectral variations in multilayer OLED structures are often strongly governed by recombination zone redistribution and interfacial excitonic interactions. We further investigated the effect of emissive layer (EML) thickness on recombination zone movement and the formation of interface-related excitons and spectral shoulder features^30^. While these studies demonstrated the utility of EPS for probing exciton dynamics under electrical operation, they were limited to specific device configurations and operating conditions.

Building upon these earlier efforts, the present work generalizes broadband EPS into a robust, bias-dependent diagnostic framework, in which the mechanistic origin of the observed parasite exciton dynamics is systematically elucidated through ultrafast TRPL measurements on a series of well-controlled thin film samples. This combined approach enables spectrally resolved, layer-by-layer characterization of excitonic degradation pathways in operating OLEDs, extending the applicability of broadband operando spectroscopy from case-specific observations to a generalized tool for diagnosing degradation mechanisms.

Here, we integrate impedance spectroscopy (IS) with operando EPS to investigate defect formation and degradation mechanisms in green OLEDs. OLED performance often declines over time due to factors like thermal instability and imperfect layer deposition, which can introduce defects and disrupt device operation^31–33^. Simultaneous electrical and optical monitoring with IS and EPS enables identification of developing defects and degradation sources. As a diagnostic tool, operando EPS elucidates internal processes impacting OLED stability and performance to advance next-generation displays. Because EPS distinguishes excitonic populations through their spectroscopic and kinetic signatures rather than relying on a specific radiative mechanism, the method is broadly applicable to phosphorescent, thermally activated delayed fluorescence (TADF), radical-based, and hot-exciton emitters.

## Results

**Fig. 1:**
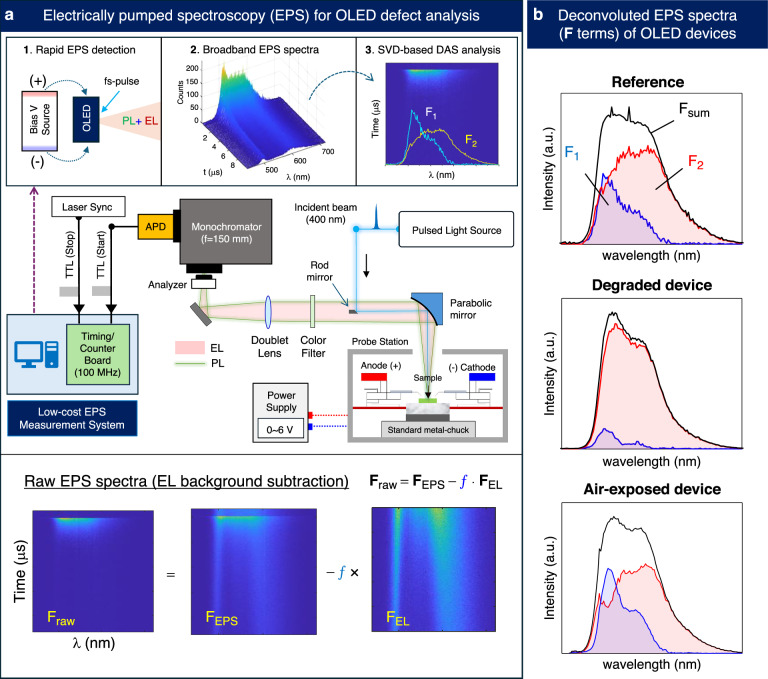
Schematic of the operando EPS framework and analysis procedure. **a** Schematic of the operando EPS setup and preparation of the raw (EL-free) EPS spectra (**F**_**raw**_) by subtracting the EL background (**F**_**EL**_) with a scaling factor (*f*). **b** Time-integrated decay-associated spectra (**F** = **A** × **D**) for reference, degraded, and air-exposed devices, highlighting differences in parasitic exciton contributions (**F**_2_, red-shaded term)

Figure 1a illustrates the operando EPS setup for real-time analysis of OLED degradation. This measures in-situ two-dimensional time-resolved photoluminescence (TRPL) spectra of the OLED device under continuous external bias voltage. The OLED device is mounted on a standard metal chuck and connected to a power supply to replicate operational conditions. A weak pulsed laser (a few pJ, 100 kHz, and <100 fs width) is employed to generate photo-induced charge carriers within the device, which can interact with excess carriers from the external source. One can then resolve the effect of electric fields on various excitonic recombination processes, including those of localized excitons in the emission layer, as well as parasite excitons formed across multi-layers. Light emitted from the OLED is directed through various optical components, including a monochromator, enabling acquisition of the complete PL spectra with high resolution. A time-correlated single photon counting (TCSPC) setup, utilizing a low-cost timer/counter board (NI-PCI-6612, 100 MHz base clock), allows rapid acquisition of long-lived PL signals from the OLED emission layer. This enables independent monitoring of both electrical and optical signals under controlled experimental conditions, capturing the continuous electroluminescence (EL) background (**F**_**EL**_), as well as the TRPL spectra (**F**_**EPS**_) obtained through EPS in real time.

The electrical analysis relies on IS to monitor variations in the electrical characteristics of the OLED, which can reveal defects or degradation within the device layers. This technique helps identify resistance and capacitance changes associated with aging or defect formation. For greater precision than electrical analysis alone can provide, we conducted chemometric analysis for the 2D-EPS spectra. To analyze the excitonic dynamics captured in the EPS data, a singular value decomposition (SVD)-based spectral analysis was employed^26^. A schematic and detailed description of the method is provided in Fig. S1.

This system was used to measure three types of devices: reference (pristine) devices, degraded devices, and air-exposed devices. The reference device serves as a control, providing baseline data for emission and impedance characteristics. The degraded device has undergone operational stress or intentional degradation treatments to simulate long-term usage and elucidate failure mechanisms. The air-exposed devices were exposed to environmental factors, such as oxygen and moisture, to study the impact of external elements. Figure 1b shows emission spectra from the three devices, each composed of two deconvoluted **F** components (**F**_*i*_ = **A**_*i* _× **D**_*i*_, *i* = 1,2). It was previously reported that **F**_1_ and **F**_2_ originate from excitonic recombination of target excitons in the EML and parasite excitons across the multilayer, respectively^26^. Since artificial degradations induce distinct changes in the populations of these two components, their intensity ratio (**F**_2_/**F**_1_) indicates device stability and efficiency losses over time. Comparison of these three devices illustrates how different degradation modes—operational stress, environmental exposure, and controlled conditions—affect OLED stability and excitonic dynamics. Before integrated analysis, we establish criteria for the performance of the degraded device relative to the reference. This allows us to set standards for degradation and validate our analysis framework before deeper electrical diagnostics.

### IS analysis of reference and degraded OLED devices

**Fig. 2:**
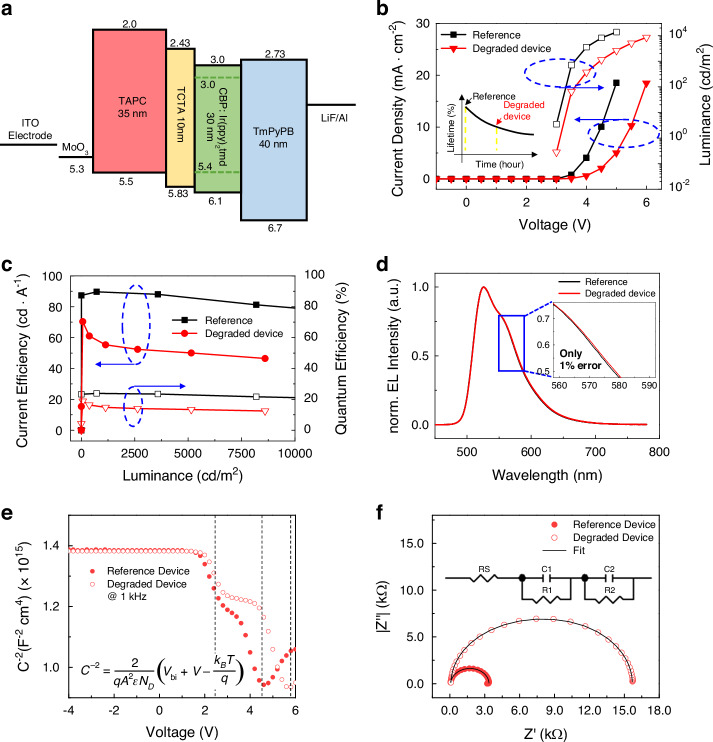
Device structure, comparative performance, and impedance analysis of reference and degraded OLEDs. **a** Energy band diagram of the OLED structure. **b** Current density–voltage–luminance (*J–V–L*) characteristics of the reference and degraded devices. **c** Current efficiency and external quantum efficiency (EQE) versus luminance, showing performance deterioration following degradation. **d** Normalized EL intensity spectra, highlighting a difference of only 1% in shoulder peak wavelength. **e** Mott–Schottky plot extracted from IS measurements. **f** Impedance spectra (Nyquist plots) under various bias conditions, illustrating increased resistance and trap-induced behavior in the degraded device

Figure 2 presents the device structure and performance metrics, focusing on the effects of degradation. Figure 2a illustrates the multilayer structure of the OLED devices: a 5 nm MoO_3_ layer; a 35 nm 1,1-Bis[(di-4-tolylamino)phenyl]cyclohexane (TAPC) hole transport layer (HTL); a 10 nm 4,4’,4”-Tris(carbazol-9-yl)triphenylamine (TCTA) exciton blocking layer (EBL); a 30 nm 4,4’-Bis(N-carbazolyl)-1,1’-biphenyl (CBP) doped with Ir(ppy)_2_tmd as the emissive layer (EML); and a 40 nm TmPyPB electron transport layer (ETL). This configuration is the same for reference (pristine) and degraded devices. In this study, CBP is employed as a representative host to demonstrate how operando EPS can resolve layer-specific degradation pathways; although CBP is used as the model system, the analytical approach and the qualitative degradation signatures identified here are applicable to more thermally robust host materials as well.

Figure 2b shows the current density–voltage–luminance (*J–V–L*) characteristics. The reference device illustrates standard performance under normal operating conditions, while the inset compares a pristine device with a degraded device driven at 2000 nits for 30 min. The degraded device exhibits delayed current injection and reduced luminance, indicating performance decline. At the same voltage, the degraded device has lower luminance and current density than the reference, suggesting that internal degradation is impairing charge injection and recombination, leading to efficiency losses. Figure 2c displays current efficiency and quantum efficiency versus luminance. Similar to the *J–V–L* trends, the degraded device demonstrates reduced efficiencies, likely due to increased non-radiative recombination and other losses in the device layers. This decrease in efficiency aligns with the observed delays in current injection, confirming how device performance declines over time from operational stress. The EL intensity graphs for the two devices (Fig. 2d) show similar shapes, the difference of only 1% making it difficult to pinpoint the specific source of degradation based on EL intensity alone. Air-exposed devices exposed to ambient air during deposition were also evaluated. As shown in Fig. S2, it exhibits minor shifts in turn-on voltage and efficiency, indicating milder deterioration than the degraded device.

Mott–Schottky and impedance spectroscopy analysis highlighted degradation-induced electronic changes in the OLED. Figure 2e presents the Mott–Schottky plots. The degraded device exhibits a steeper slope in the 1/*C*^2^ versus voltage plot compared to the reference, resulting in a lower apparent carrier concentration and a reduced built-in potential (*V*_bi_). In the voltage range 3.0−4.5 V, *V*_bi_ of the degraded device is **~**0.43 V lower than the reference, implying enhanced charge trapping at interfacial sites and reduced net carrier density. Figure 2f shows the impedance spectra. The degraded device displays an increased impedance, suggesting increased trap states and charge accumulation. This can be partially interpreted by modeling the device as a simplified p–n junction, where the capacitance and resistance components (*C*_1_||*R*_1_ and *C*_2_||*R*_2_) correspond to the p-type (TAPC, TCTA) and n-type (CBP:Ir(ppy)_2_tmd, TmPyPB) regions, respectively^34–37^. Impedance analysis based on this model provides the resistance and capacitance values for each region. However, pinpointing the individual layer(s) responsible for degraded performance is difficult due to overlapping electrical responses across interfaces, as discussed in Fig. S3. We therefore propose an integrated analysis with operando EPS for deeper insight.

### EPS analysis of reference and degraded OLED devices

**Fig. 3:**
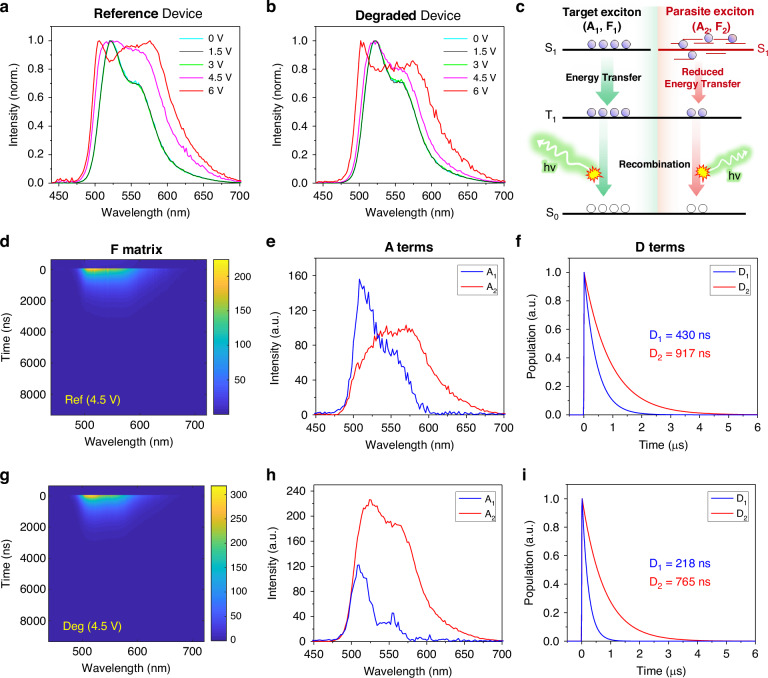
Steady-state EPS spectra and decay-associated spectral (DAS) analysis of reference and degraded OLEDs. **a**, **b** Operando EPS spectra of the reference and degraded devices under increasing bias voltage (0–6 V). **c** Schematic illustration for the formation of target and parasitic excitons. **d**, **g** DAS components at 4.5 V for the reference and degraded devices, respectively, decomposed into (**e**, **h**) **A** (principal spectrum) and (**f**, **i**) **D** (population decay) terms, showing degradation-induced changes in exciton decay dynamics

While the electrical analysis suggests potential sites of degradation, we conducted operando EPS measurements on the two devices in a more comprehensive investigation to localize degradation effects, as shown in Figs. S4 and S5. Figure 3a and b present the time-integrated EPS spectra of the reference and degraded devices, respectively. Under zero bias, both devices show nearly identical dopant emission profiles. At the turn-on voltage, however, the spectral width is broader in the reference device, suggesting a difference in recombination environments. However, steady-state spectra alone are insufficient to quantitatively distinguish the contributions of parasitic and emissive excitons. Figure 3c illustrates the conceptual difference between target excitons (**A**_1_) contributing to desired emission and parasitic excitons (**A**_2_) involved in non-radiative or interface-related processes. EPS data were further analyzed using decay-associated spectra (DAS) analysis as outlined in Fig. 1b. Representative *F*-matrix results for 4.5 V are shown in Fig. 3d and g, with corresponding **A** terms in Fig. 3e and h, and **D** terms in Fig. 3f and i for reference and degraded devices, respectively. The full DAS results, including all **A** and **D** terms, are summarized in Figs. S6 and S7.

**Fig. 4:**
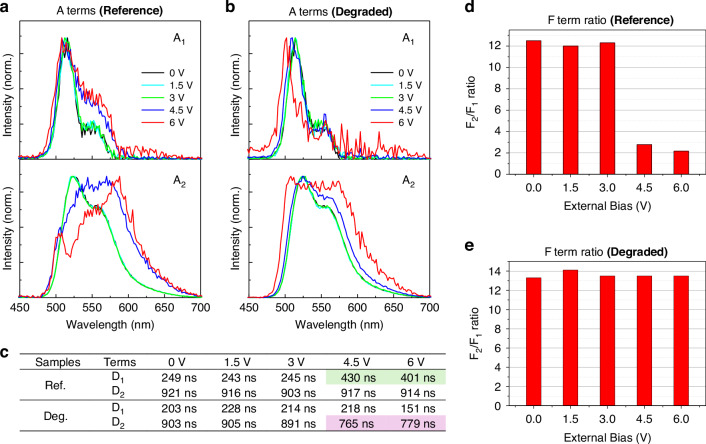
Voltage-dependent spectral and temporal behavior of target and parasitic excitons in reference and degraded OLEDs. **a**, **b** Normalized principal spectra (**A** terms), and **c** corresponding population relaxation times (time-constants for the **D** terms). **d**, **e** Intensity ratio (**F**_2_/**F**_1_) between target and parasite excitonic emissions for the two devices

The spectral and kinetic behavior of excitons exhibited clear differences between reference and degraded devices. Two principal components, **A**_1_ and **A**_2_, corresponding to localized target excitons in the EML and parasitic excitons formed at multilayer interfaces, respectively, were compared. Under increasing bias, the **A**_1_ term grew prominently in the reference device, while remaining nearly constant in the degraded one, indicating that efficient charge migration to the EML occurs only in the reference device, due to the formation of localized target excitons. In the degraded OLED, however, the **A**_1_ component exhibits a slight blue shift at 6 V, which reflects voltage-induced exciton redistribution toward locally higher-energy regions within the damaged CBP host matrix. The formation of ultrafast non-radiative scattering sites limits charge migration into the EML, causing the remaining emissive excitons to occupy less perturbed domains under high bias. In the reference device, the **A**_1_ term suddenly changed shape at the turn-on voltage, showing a relatively strong 0–1 vibronic peak around 550 nm (Fig. 4a, upper), while its lifetime (**D**_1_ term) further increased from about 245 to 401–430 ns (Fig. 4c). The enhanced second vibronic peak provides evidence that Ir complexes embedded in a pristine CBP matrix can undergo substantial structural relaxations under external electric fields. These relaxed Ir complexes are responsible for the prolonged PL lifetime of the **D**_1_ term. In contrast, for the degraded device, both the **A**_1_ and **D**_1_ terms were relatively insensitive to the bias voltage (Figs. 4c and S7). This may indicate that a degraded CBP matrix, which inhibits charge migration into the EML, facilitates the formation of parasite excitons near the layer interface.

Meanwhile, the **A**_2_ term for the reference device exhibited a clear redshift and asymmetric spectral broadening as the bias increased (Fig. 4a, bottom), suggesting strong excitonic interactions at the interfaces between the EML and adjacent layers. In contrast, the **A**_2_ term of the degraded device remained nearly symmetric in shape with relatively weak spectral broadening (Fig. 4b, bottom). Under high bias (6 V), however, the **A**_2_ component undergoes pronounced spectral broadening, which reflects enhanced interfacial excitonic coupling after degradation. Our DAS and SAS analyses indicate that morphological relaxation in the CBP host and increased exciton accumulation at the EML/EBL boundary promote stronger mixing between host- and dopant-associated emission pathways, giving rise to this broadened spectral signature. This difference extended to the **D**_2_ terms, with the degraded device showing relatively fast relaxation compared to the **D**_2_ lifetimes observed in the reference device, as summarized in Fig. 4c. This indicates that interfacial excitonic interactions leading to the formation of parasite excitons are relatively stronger in the reference device than in the degraded device. This is observed even though the spectral contribution of the **A**_2_ term is significantly reduced under higher bias in the reference device (see Fig. S6d and e). We can infer that artificial thermal degradation weakens the interfacial excitonic interactions between the EML and its adjacent layers, disrupting efficient charge and energy transfer pathways.

The first and second components were extracted from the relation **F** = **A** × **D**, as shown in Fig. S8. The intensity ratios (**F**_2_/**F**_1_) for reference and degraded devices are plotted in Fig. 4d and e, respectively. The reference device exhibited a sharp decline in this ratio near the turn-on voltage, indicating a rapid suppression of parasitic exciton contributions under operating conditions. In contrast, the degraded device showed a nearly constant **F**_2_/**F**_1_ ratio across the entire voltage range. This reflects the disruption of exciton selectivity and recombination dynamics caused by degradation. The **F**-ratio serves as a simple quantitative metric for identifying device degradation and excitonic imbalance in multilayer OLEDs.

While the EPS-based DAS analysis identified the EML as the primary region affected by degradation, the detailed mechanistic pathways underlying exciton recombination and interfacial scattering remain unclear. To further investigate and pinpoint the origin of performance loss, we fabricated a series of samples by systematically degrading each functional layer within the OLED structure. To isolate the layer most responsible for the observed decline in performance, we conducted picosecond TRPL measurements on both single-layer and bilayer configurations that replicate the layer architecture of the full device.

### Ultrafast exciton dynamics in single and bilayer films

Steady-state and time-resolved PL measurements on individual layers of the device are shown in Figs. S9−S13. In the single-layer EML (CBP:Ir) film, the steady-state dopant emission at ~525 nm was significantly quenched with increasing temperature, as shown in Fig. S9a, suggesting impaired energy transfer within the EML. This observation was supported by picosecond TRPL spectral measurements and corresponding TRPL profiles at 560 nm (Figs. S10 and S11), where CBP:Ir–150 showed a shortened dopant lifetime and diminished emission.

TAPC and TmPyPB exhibited irregular, broad, and variable PL spectra, attributed to their inherent conformational distribution of flexible side groups^38^, while TCTA remained largely unchanged, consistent with its rigid molecular structure (Fig. S9b–d). Nonetheless, both TCTA and TAPC films maintained similar average lifetimes across degradation levels (Figs. S12 and S13), confirming that their degradation effects are minor. The PL spectra of TmPyPB were found to be highly sensitive to fabrication conditions (Fig. S9d). Bilayer films were therefore employed for subsequent time-domain analysis.

Further analysis, using time-resolved area-normalized emission spectra (TRANES), on the TRPL spectra of CBP:Ir (Fig. S14) revealed clear iso-emissive points near 480 nm, indicative of direct host-to-dopant energy transfer^39^. However, in degraded samples, the narrowed CBP emission and suppressed dopant PL signal imply exciton scattering by damaged host sites. These results collectively identify the EML as the primary degradation site, prompting further analysis of excitonic processes involving CBP:Ir. Although the degradation signatures identified here—such as emission narrowing and delayed host–dopant energy transfer—are presented using CBP as the model host, these behaviors reflect general morphological relaxation processes that have also been observed in more thermally robust host systems. CBP therefore serves as an effective representative platform for demonstrating how operando EPS can resolve host-related degradation mechanisms with layer-level specificity.

**Fig. 5:**
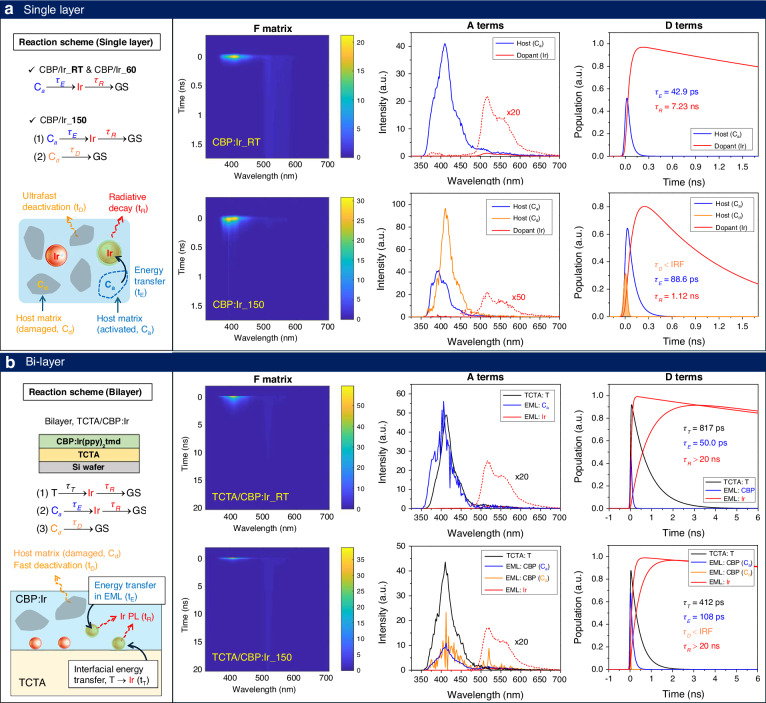
Layer-resolved kinetic modeling and SAS analysis of degraded OLED structures. Kinetic modeling and species-associated spectra (SAS) analysis of CBP:Ir-based **a** single-layer and **b** bilayer films at room temperature (RT) and thermal degradation (150 °C) conditions. Reaction schemes used in the SAS analysis are shown in the left panels

We applied species-associated spectra (SAS) analysis to the TRPL data of CBP:Ir films under different thermal conditions, using the predefined kinetic schemes illustrated in Fig. 5a. (The full SAS results, including CBP:Ir–**60**, at 60 °C, are provided in Fig. S15.) For CBP:Ir−**RT** (reference), the TRPL spectra were well-fitted using a serial energy transfer model (**C**_a_ → **Ir** → **GS**), where **C**_a_ denotes the activated CBP host, **Ir** the dopant, and **GS** the ground state. Both films exhibited fast energy transfer (*τ*_E_ ≈ 43 ps) followed by slow excitonic recombination (*τ*_R_ > 4 ns), consistent with an intact EML environment. In contrast, the highly degraded film (CBP:Ir_150) required the inclusion of an ultrafast exciton quenching pathway (**C**_d_ → **GS**), where **C**_d_ represents the damaged CBP matrix. Although the **A** term of **C**_d_ in the degraded CBP:Ir_150 film appears similar in spectral shape to **C**_a_ in the pristine CBP:Ir_RT film due to their shared CBP origin, **C**_d_ represents a fundamentally different excitonic pathway. Its IRF-limited lifetime and strong non-radiative quenching behavior indicate that **C**_d_ arises from highly disordered or damaged CBP sub-domains created during thermal stress, rather than from the normal host-relaxation process described by **C**_a_. This additional decay channel was characterized by an intense **A** term, with a near-instantaneously quenched **D** term with an instrument response function (IRF)-limited lifetime (*τ*_D_ < *τ*_IRF_). Degradation weakened the electronic coupling between host and dopant, increasing energy transfer time (≈43 ps → ≈89 ps), and reducing the dopant PL lifetime. These results confirm that thermal degradation of the CBP matrix introduces non-radiative scattering sites that impair both host-to-dopant energy transfer and radiative exciton recombination. Notably, the spectral and kinetic signatures associated with this process—such as the emergence of ultrafast exciton-scattering channels and broad host-emission features—indicate glass-transition-like morphological relaxation rather than chemical bond cleavage, as no signatures of bond-breaking-induced species were observed.

To replicate the device configuration, bilayer films (TCTA/CBP:Ir, CBP:Ir/TmPyPB, and TAPC/TCTA) were fabricated with thicknesses identical to those used in the actual OLEDs. As shown in Figs. S16 and S17, their steady-state PL spectra exhibited significant convolution between the constituent layers. In particular, the TCTA/CBP:Ir bilayer showed enhanced dopant emission compared to the single-layered EML, suggesting that the TCTA overlayer effectively reduces non-radiative exciton scattering at the EML’s exposed surface. This enhancement implies possible interfacial excitonic interactions that may influence device performance. To investigate this possibility, TRANES analysis was performed on the TCTA/CBP:Ir bilayers under reference and degraded conditions. As shown in Fig. S18, an iso-emissive point emerged consistently around 480 nm, indicating a directional population transfer from TCTA to the Ir dopant. Notably, the PL band originating from TCTA (~410 nm) remained spectrally stable over long time-delays (>0.5 ns), even in the TCTA/CBP:Ir−**150** bilayer, confirming that the observed spectral evolution arises from interfacial energy transfer rather than from changes in intrinsic exciton relaxation pathways of the individual layers. These results confirm that interfacial excitonic coupling is particularly significant at the TCTA/EML junction, where energy transfer from the EBL to the dopant can occur. Such interfacial processes may account for the broad and asymmetric excitonic bands observed in the DAS analysis of the full device.

To quantify the interfacial exciton transfer dynamics revealed in the TRANES analysis, kinetic modeling incorporating interfacial energy transfer (**T** → **Ir** → **GS**) was performed on the TRPL spectra of the TCTA/CBP:Ir bilayer films, as indicated in the left panel of Fig. 5b. (The full SAS results, including TCTA/CBP:Ir−**60**, are provided in Fig. S19.) Under reference conditions, the spectral components associated with the TCTA layer (**T**, donor) and Ir dopant (acceptor) were clearly deconvoluted, allowing extraction of the interfacial energy transfer time (*τ*_T_). As summarized in Fig. S19, SAS fitting resolved the overlapping PL bands from both TCTA and CBP, and the associated **D** terms revealed a slow interfacial exciton transfer (*τ*_T_ ≈ 820 ps) within the EML. Importantly, compared to the corresponding single-layer films, the energy transfer time (*τ*_E_) from CBP host to Ir dopant remained largely unaffected. Following degradation, however, *τ*_T_ decreased to ~400 ps in the 150 °C-annealed film, indicating that interfacial excitonic coupling can be locally enhanced at degraded sites due to morphological or energetic disorder. While Ir-based dopants can also act as hole-trapping centers in some systems, potentially contributing to localized recombination and Joule-heating-induced degradation, our SAS and DAS results suggest that the dominant interfacial signatures observed here arise primarily from CBP matrix relaxation and enhanced interfacial coupling rather than direct dopant-mediated trapping. Because these interfacial exciton-redistribution processes arise from general exciton–matrix interactions rather than CBP-specific chemistry, the same mechanistic principles are expected to apply in exciplex co-host EMLs, where the P-type component effectively functions as an exciton-blocking layer. This accelerated transfer may facilitate exciton accumulation near the EBL/EML boundary, thereby contributing to the increased parasitic exciton generation observed in degraded devices. These observations are consistent with the enhanced **A**_2_ component and faster decay kinetics observed in the DAS analysis of the degraded OLEDs (see Fig. 4), further supporting the role of the interface in the formation of parasitic excitons under stressed conditions.

To confirm that excitonic interactions occur specifically at the interface of the EML, bilayer combinations such as CBP:Ir/TmPyPB and TAPC/TCTA were examined. These films exhibited negligible correlation in spectral evolution or interfacial energy transfer. This supports the hypothesis that strong excitonic coupling is specific to the TCTA/CBP:Ir configuration, as shown in Figs. S20 and S21. Air-exposed OLEDs structure incorporating a spacer layer between EML and EBL was fabricated to spatially decouple interfacial interactions. As shown in Fig. S22, the **A**_2_ component associated with parasite excitons was significantly suppressed, directly confirming the critical role of the EBL/EML interface in degradation-induced excitonic scattering.

### Proposed mechanisms behind OLED device degradation

The exciton dynamics in the CBP:Ir single-layer and TCTA/CBP:Ir bilayer films were modeled using the kinetic schemes introduced in Fig. 5. In the single-layer case, energy transfer occurs from the active CBP host to the Ir dopant, followed by radiative decay, while thermal degradation introduces a rapid non-radiative pathway via damaged host sites. The bilayer configuration introduces an additional interfacial exciton transfer from TCTA to Ir, enabling the extraction of the interfacial transfer time constant (*τ*_T_). Under degraded conditions, *τ*_T_ was notably shortened, suggesting that excitonic coupling is locally enhanced at the disordered EBL/EML boundary, promoting the generation of parasitic excitons not observed in the single-layer films. Building on these mechanistic insights, a schematic illustration is proposed to summarize the overall degradation pathways.

**Fig. 6:**
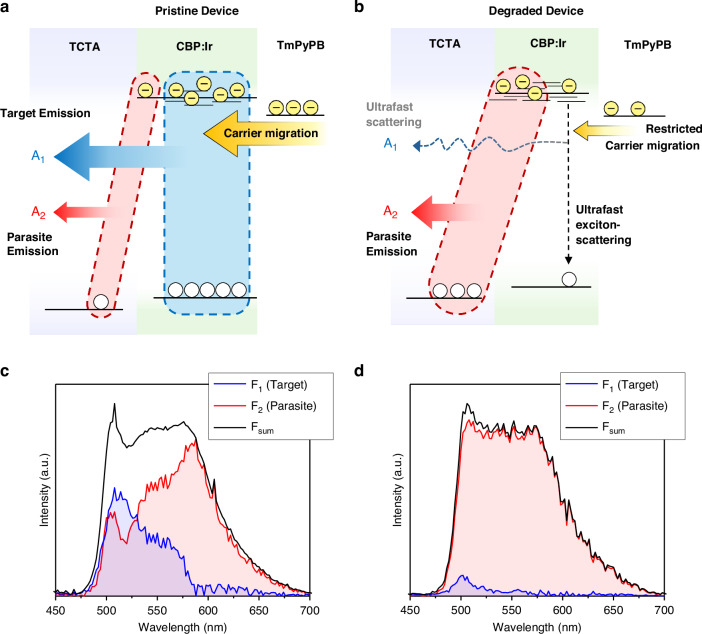
Schematic illustration of exciton recombination and parasitic exciton scattering revealed by operando analysis. **a**, **b** Schematic illustrations and **c**, **d** representative DAS results (**F** = **A** × **D**) depicting recombination and scattering pathways in reference (pristine) and degraded OLEDs, respectively, under 6 V bias. The red-shaded region highlights the increased contribution of parasitic excitons (**F**_2_ term) in the degraded sample. The blue-shaded region corresponds to target exciton emission (**F**_1_ term)

Figure 6 illustrates exciton dynamics in reference and degraded OLED devices under operational bias. In the reference device (Fig. 6a), the applied electric field promotes efficient formation of target excitons within the emissive layer (EML), leading to strong dopant emission represented by the **F**_1_ term. Moderate parasite exciton emission (**F**_2_ term) arises from interfacial coupling between the EML and EBL (TCTA), but most weakly bound excitons dissociate under the field, thereby contributing to the population of target excitons (Fig. 6c). As a result, only strongly coupled interfacial excitons persist near the interface, manifesting as a broad and asymmetric spectral feature in the **F**_2_ component.

In the degraded device (Fig. 6b), thermal damage to the CBP host leads to the formation of ultrafast non-radiative scattering sites. These sites quench excitons before radiative recombination can occur, effectively shifting the recombination zone toward the EML/EBL interface. This shift enhances the **F**_2_ component and suppresses the **F**_1_ component, reflecting increased exciton loss and reduced radiative efficiency (Fig. 6d). In addition, the strengthened interfacial excitonic interaction, as evidenced in Fig. S19, may facilitate the confinement of excitons at the damaged interface, further promoting parasitic recombination pathways. Moreover, thermal degradation may also compromise charge transport. As shown in Fig. S20, degraded TmPyPB exhibited ultrafast exciton deactivation (*τ*_m_ ≈ 92 ps), indicating that electron transport is increasingly hindered in the ETL, potentially competing with the recombination and scattering processes. Although TmPyPB exhibits intrinsic thermal instability, our EPS and TRPL analyses show that its degradation contributes only weakly compared to the dominant pathways originating from host-matrix relaxation within the EML and exciton scattering at the EML/EBL interface. Together, these findings present a comprehensive degradation mechanism involving both excitonic and electronic disruptions that collectively cause deterioration in device performance. These mechanistic insights suggest that hosts with greater resistance to morphological relaxation, together with EML/EBL combinations that reduce interfacial exciton accumulation, are more suitable for achieving thermally stable and long-lived OLEDs.

The development of operando electrically pumped spectroscopy (EPS), combined with kinetic and SAS analysis, has provided a powerful method for identifying internal degradation pathways that are difficult to detect through conventional electrical measurements alone. Here, EPS revealed subtle spectral changes linked to interfacial excitonic coupling and EML degradation, while SAS analysis of time-resolved PL data from controlled single- and bilayer films pinpointed the origin of parasitic exciton formation.

## Discussion

This study presents a comprehensive investigation into the degradation mechanisms of OLED devices using operando EPS in conjunction with TRPL and advanced kinetic modeling techniques. While conventional electrical measurements provide limited insight into the microscopic origins of efficiency loss, our approach enabled layer-resolved and interface-specific optical analysis under operating conditions. Our results demonstrate that degradation primarily occurs within the EML, where thermal stress induces structural damage to the CBP host matrix. This damage introduces ultrafast exciton scattering sites that disrupt both host-to-dopant energy transfer and radiative recombination, as confirmed by SAS analysis. In addition, DAS of the full device revealed increased contributions from parasitic excitons, which become confined near the interface between the EML and adjacent layers under electric bias.

Bilayer experiments further elucidated the role of interfacial excitonic coupling, particularly at the EML/EBL boundary (TCTA/CBP:Ir), where directional energy transfer from the EBL to the dopant was observed. Following degradation, the interfacial coupling was locally enhanced, contributing to spectral broadening and asymmetric emission features. These effects were not observed in control bilayers lacking such coupling, nor in air-exposed devices designed to suppress interface interactions, thereby isolating the EML/EBL interface as a critical site of degradation-induced excitonic scattering. By integrating EPS, TRPL, and SAS/DAS analysis across controlled single-layer and bilayer films, we constructed a unified mechanistic picture of degradation. This framework accounts for both spectral and temporal evolutions of exciton populations and offers valuable diagnostic indicators for monitoring OLED reliability. These mechanistic insights indicate that employing hosts with greater resistance to morphological relaxation, or using co-host systems designed to suppress such relaxation, along with EML/EBL combinations that minimize interfacial exciton accumulation, can provide a more stable excitonic environment for long-term device operation. Overall, this work highlights the power of operando EPS-based analysis in visualizing defect formation and exciton behavior in real time, providing critical insights for designing stability-enhancing strategies in current and future OLED technologies.

## Materials and methods

### Substrate cleaning and OLED device fabrication

Glass substrates were sequentially cleaned by sonication in acetone and isopropyl alcohol for 10 min each, followed by nitrogen drying. All organic and inorganic layers were deposited using thermal evaporation under a base pressure of 2.0 × 10^−7^ Torr. 1,1-Bis[(di-4-tolylamino)phenyl]cyclohexane (TAPC, used as the hole transport layer, HTL) and tris(4-carbazoyl-9-ylphenyl)amine (TCTA; used as the exciton blocking layer, EBL) were deposited with thicknesses of 35 and 10 nm, respectively. The emissive layer (EML) consisted of a co-deposited mixture of CBP doped with 8 wt% bis(2-phenylpyridine)(tetramethyl-1,3-dioxolane) iridium(III) (Ir(ppy)_2_tmd) (30 nm). Ir(ppy)_2_tmd is a green phosphorescent dopant widely used in CBP-based EMLs due to its high radiative decay rate and favorable host-to-dopant energy-transfer compatibility. A doping concentration of 8 wt% was selected to ensure efficient Förster-type energy transfer while suppressing concentration quenching, consistent with commonly adopted CBP-based phosphorescent OLED architectures. Following the EML, a 40 nm-thick TmPyPB layer was deposited as the electron transport layer (ETL). Finally, lithium fluoride (LiF, 1 nm) and aluminum (Al, 100 nm) were thermally evaporated as the cathode using a shadow mask defining an active area of 4 mm^2^, with deposition rates of 0.5 and 3 Å/s, respectively. Current density–voltage–luminance (*J*–*V*–*L*) and electroluminescence (EL) measurements were performed using a Keithley 2400 source meter and a Minolta CS-2000 spectroradiometer.

### Time-resolved photoluminescence

The light source was an in-house-built cavity-dumped Ti:sapphire oscillator with a center wavelength of 800 nm. The output pulse energy was 30−40 nJ at a variable repetition rate of 17−100 kHz. Excitation pulses at 266 nm were generated by an in-house-built noncollinear tripler using 100 μm thick BBO (β-barium borate) crystals. A parabolic mirror was used to focus the excitation beam onto the device sample, and the EPS signal was collected in a backscattering geometry using the same mirror. For EPS measurements, OLED devices were driven at 3–6 V from a DC power source. The PL and EL emissions were directed to a monochromator (SP2150, Princeton Instruments) and detected with an avalanche photodiode (ID-100-50, IDQ Inc.). Fast EPS measurements were performed using a 100 MHz timing/counter board (PCIe-6612, National Instruments). For picosecond TRPL spectral measurements, a commercial time-correlated single-photon counting board (SPC-130-EMN, Becker & Hickl Inc.) was used to record TRPL profiles directly with ~50 ps time resolution. All the instruments were controlled in unison by custom-designed LabVIEW software, allowing the recording of steady-state and time-resolved emission spectra directly.

### Electrical analysis

Impedance spectroscopy (IS) and *C–V* measurements were performed using an HP 4284A precision LCR meter. For IS, the frequency was swept from 25 Hz to 1 MHz with a 45 mV_rms_ AC modulation and a 6 V DC bias. Nyquist plot modeling and fitting were conducted using ZView software (Scribner Inc.). *C–V* measurements were carried out by varying the DC bias from 0 to 8 V, while applying a 45 mV_rms_ AC modulation at 1 kHz.

## Supplementary information

Supplementary Information

## Data Availability

Data that support the plots presented in this paper and other findings of this study are available from the corresponding author upon reasonable request.
