# Supplementary material for: Investigating degradation mechanisms in organic light-emitting diodes using operando electrically pumped spectroscopy
Source: Light Sci Appl. 2026 Jul 6;15:304. doi: 10.1038/s41377-026-02373-8 (PMC13334042; doi:10.1038/s41377-026-02373-8)
Supplement: Supplementary file 1 — Supplementary Information [file 41377_2026_2373_MOESM1_ESM.docx]

**Supplementary Information for**

**Investigating Degradation Mechanisms in Organic Light-emitting Diodes using Operando Electrically Pumped Spectroscopy**

Chang Min Lee^1,a)^, Hyun Jae Lee^1,a)^, Insung Ha^1^, Mengdi Fu^1^, Muhammad Waheed^1^, Geon Lee^1^, Deepak Rajaram Patil^1^, P. Justin Jesuraj^2,3^, Jae Woo Lee^4,b)^, Chul Hoon Kim^5,6,b)^ and Seung Yoon Ryu^1,7,b)^

^1^Department of Physics, Dongguk University, Seoul 04620, Republic of Korea

^2^Department of Physics and Nanotechnology, SRM institute of Science and Technology-Kattankulathur, Chengalpattu- 6032033, India.

^3^Centre of Excellence in Materials for Advanced Technologies (CeMAT), SRM institute of Science and Technology-Kattankulathur, Chengalpattu- 6032033, India.

^4^Education and Research Center for Artificial intelligence, Smart Convergence Technology and Department of Electronics and Information Engineering, Korea University, Sejong City 30019, Republic of Korea

^5^Department of Advanced Materials Chemistry, Korea University, Sejong 30019, Republic of Korea

^6^Division of Smart Energy Convergence Engineering, Korea University, Sejong, 30019, Republic of Korea.

^7^Photoenergy Harvesting and Conversion Technology (*phct*), Dongguk University, Seoul 04620, Republic of Korea

^a)^ C.M. Lee and H.J. Lee contributed equally to this work.

^b)^ Authors to whom correspondence should be addressed: [orion627@korea.ac.kr](mailto:orion627@korea.ac.kr), [chulhoon@korea.ac.kr](mailto:chulhoon@korea.ac.kr), [justie74@dongguk.edu](mailto:justie74@dongguk.edu)

Keyword: Operando electrically pumped spectroscopy (EPS) analysis, degradation mechanism, organic light-emitting diodes (OLEDs), exciton dynamics, layer-resolved photophysics

**
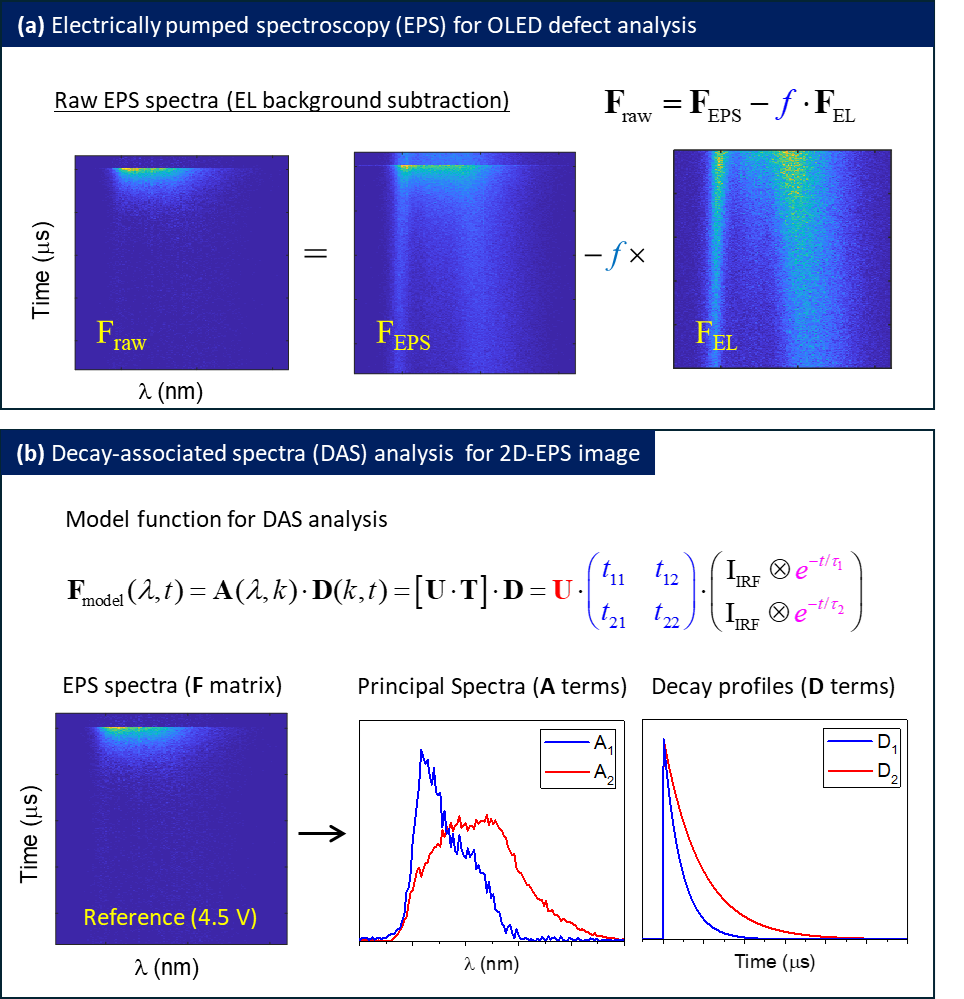
**

**Fig. S1: (a) Electrically pumped spectroscopy (EPS) for OLED defect analysis and (b) decay-associated spectra (DAS) analysis of the raw EPS spectra based on singular value decomposition.**

**Supplementary Fig. S1** presents the schematic spectral analysis procedure for extracting the principal spectral components of the EL-free electrically pumped spectroscopy (EPS) data (**F_raw_**). To exclusively monitor photo-induced events in the excitonic recombination processes, the steady-state electroluminescence (EL) background (**F_EL_**) was subtracted from the time-resolved EPS spectra (**F_EPS_**), yielding the raw EPS spectra **F_raw_**. Decay-associated spectra (DAS) analysis was then performed on **F_raw_** using singular value decomposition (SVD), which enabled decomposition into spectral (**A**) and temporal (**D**) components.^4^ The decomposition process follows the relation **F = A×D**, where the **D** terms represent population kinetics modeled using exponential functions, convoluted with the instrument response function (IRF) of the EPS setup. The **A** terms were derived by applying a square transform matrix (**T**) to the **U** vector obtained from the SVD of **F_raw_**. Nonlinear least-squares fitting was used to optimize both the **T** matrix and the kinetic parameters of the **D** terms. This approach allows extraction of spectrally distinct features corresponding to different excitonic populations and their decay dynamics, thereby providing insights into emission behavior changes induced by degradation or defect formation. The decomposition also facilitates comparison of component-wise intensities and kinetics across various device conditions.

**
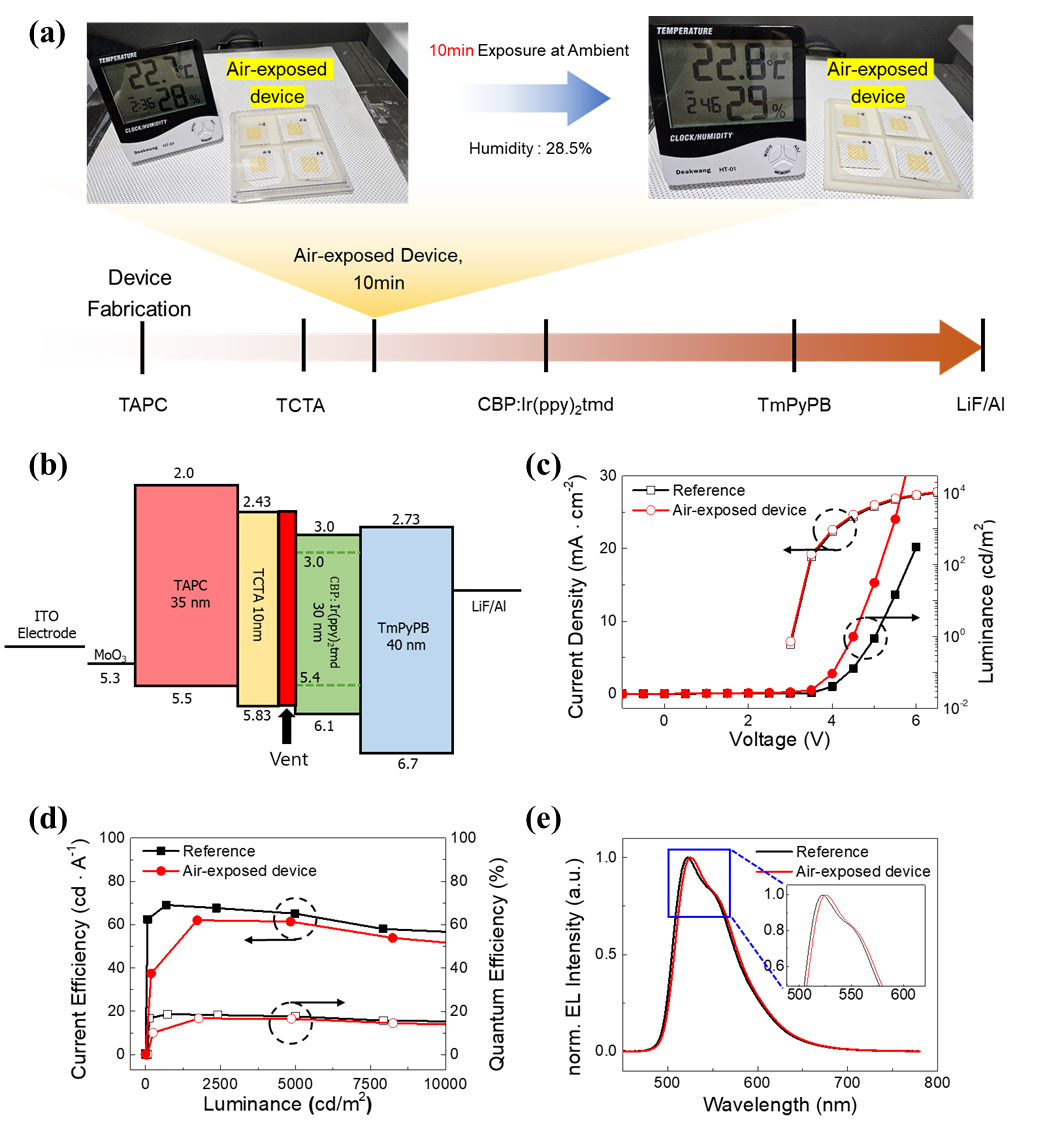
**

**Fig. S2: Environment, structure and performance of air-exposed OLEDs device exposed to ambient air for 10 minutes:** (a) environmental conditions; (b) structure schematic; (c) current density−voltage-luminance curve; (d) current and quantum efficiencies; (e) electroluminescence intensity.

**Supplementary Fig. S2** shows the characterization results of air-exposed OLED devices, which was exposed to ambient air for 10 minutes during the fabrication process. The environmental conditions (22.7−22.8 ^o^C, 28−29 % RH) under which the air-exposed devices were handled are shown in (a). The multilayer structure in (b) corresponds to the reference device, consisting of TAPC (35 nm)/TCTA (10 nm)/CBP:Ir(ppy)_2_tmd (30 nm)/TmPyPB (40 nm). The *J–V* curve in (c) (left *y*-axis) reveals a slightly delayed turn-on voltage and lower current density, suggesting minor interfacial changes caused by brief ambient exposure. Similarly, the EQE and current efficiency shown in (d) are marginally reduced across the luminance range. The EL intensity in (e) also shows a small decrease in absolute magnitude. The normalized EL spectra exhibit negligible peak shifts or spectral broadening, implying that the emissive layer remains optically stable. These results suggest that short-term ambient exposure causes mild electrical degradation, primarily affecting charge injection and efficiency. However, the core emission characteristics are largely preserved. This underscores the importance of environmental control during fabrication, even for brief interruptions. EPS analysis of the air-exposed devices was also conducted, and the results will be discussed in a later section.

**
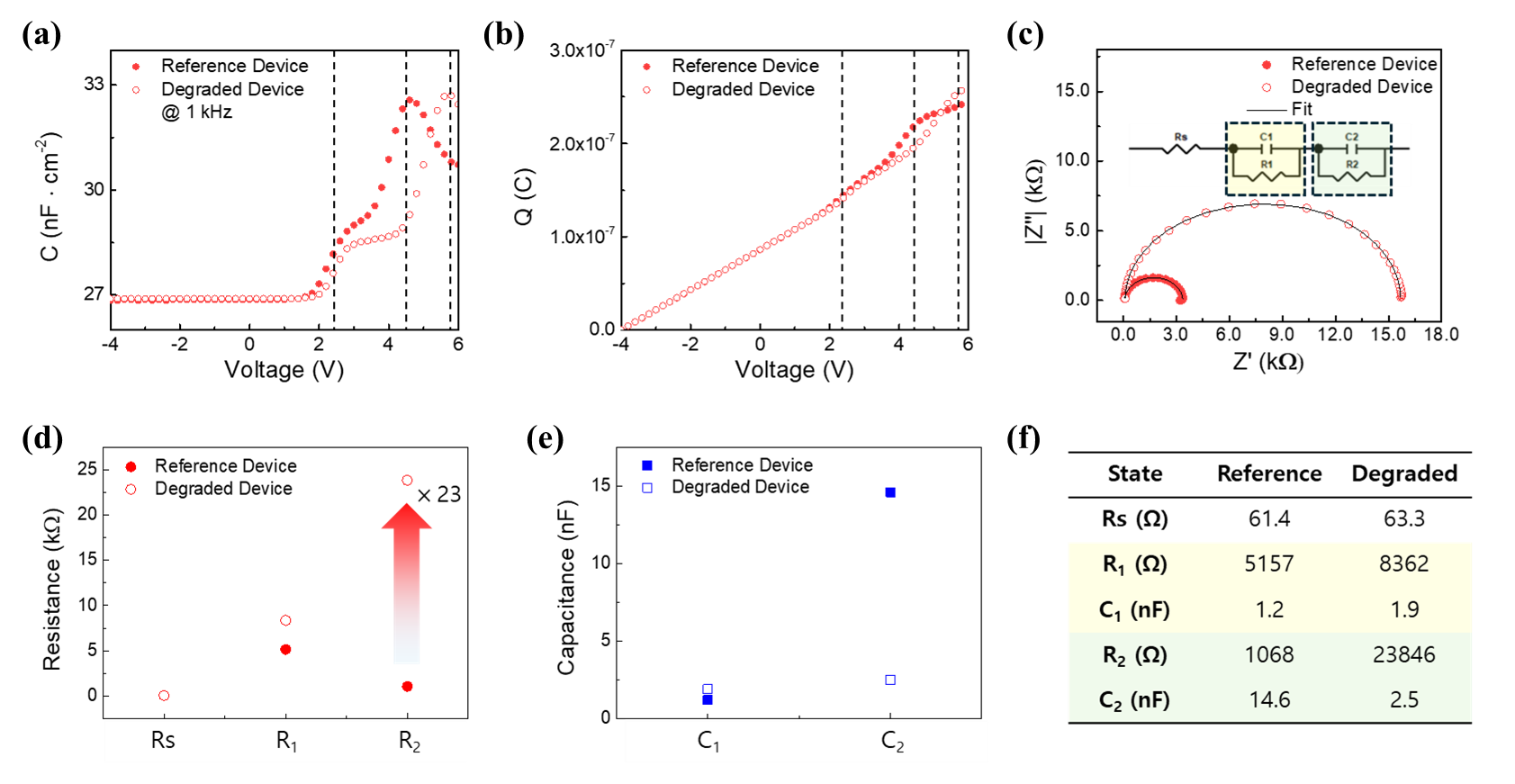
**

**Fig. S3: Impedance-based quantitative analysis of electrical degradation in OLED devices using equivalent circuit modeling.** (a) *C-V* curve; (b) *Q-V* curve; (c) impedance fitting using equivalent circuit; (d, e) equivalent resistance and capacitance; (f) *R, C* values.

**Supplementary Fig. S3** presents additional electrical characterization of reference and degraded OLED devices. **Figures S3a** **and S3b** show the capacitance–voltage (*C–V*) and total charge (*Q-V*) curves obtained from the same measurement protocol used in the main text. The degraded device exhibits reduced capacitance at low voltages (3.0–5.0 V), indicative of enhanced trap formation or interfacial defects. The corresponding *Q–V* plot shows a suppressed total charge response in the degraded device at low voltage, which suggests inhibited charge storage due to interface traps. However, at higher voltages (5.0–6.0 V), *Q* increases more rapidly in the degraded device than in the reference, indicating that trap states begin to accumulate charge once filled. This behavior implies that degradation introduces trap sites with voltage-dependent occupancy, affecting both charge injection and retention characteristics.

To quantitatively interpret these changes, impedance fitting was performed using the equivalent circuit shown in **Fig. S3c** inset, which includes a series resistance (*R*s) and two parallel *R–C* elements (*C_1_||R_1_* and *C_2_||R_2_*), corresponding to the p-type and n-type layers, respectively. As shown in **Fig. S3d and S3e**, *R_2_* increased significantly in the degraded device, reaching a value approximately 23 times higher than that of the reference. In contrast, *R_1_* and *C_1_* showed relatively small changes. This result suggests that the dominant resistance change occurs in the *R_2_* branch of the model. However, because the equivalent circuit does not directly correspond to specific physical layers, the increase in *R_2_* alone cannot conclusively identify the origin of the degradation. To clarify the physical source of these electrical changes, we conducted operando EPS analysis.


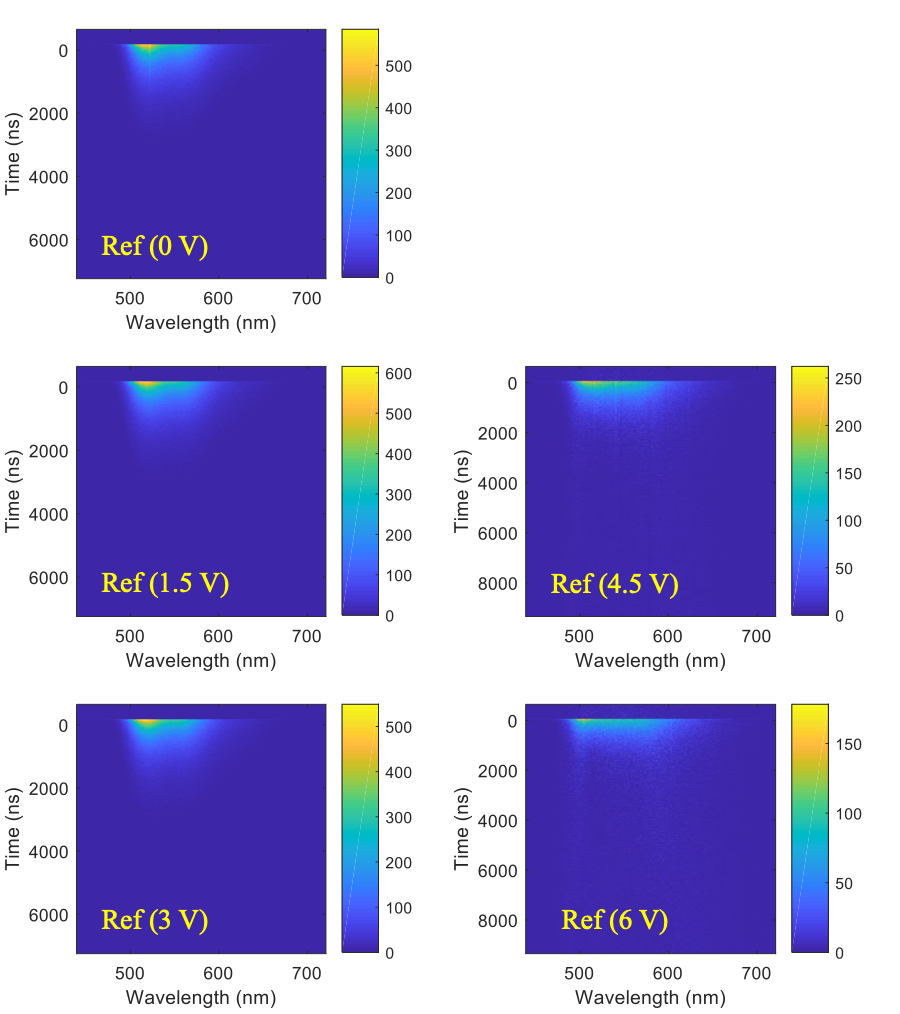


**Fig. S4: Raw EPS spectra (F_raw_) of the reference device (Ref) under various external bias voltages.**

The full set of steady-state EPS spectra for the reference OLED device, measured under various bias voltages, is presented in **Supplementary Fig. S4**. With increasing voltage from 0 V to 6 V, changes in spectral width and intensity are observed. These trends reflect the progression of excitonic recombination dynamics and the activation of additional recombination pathways, including those involving interfacial states. This dataset serves as a baseline for evaluating degradation-induced changes in degraded devices.

**
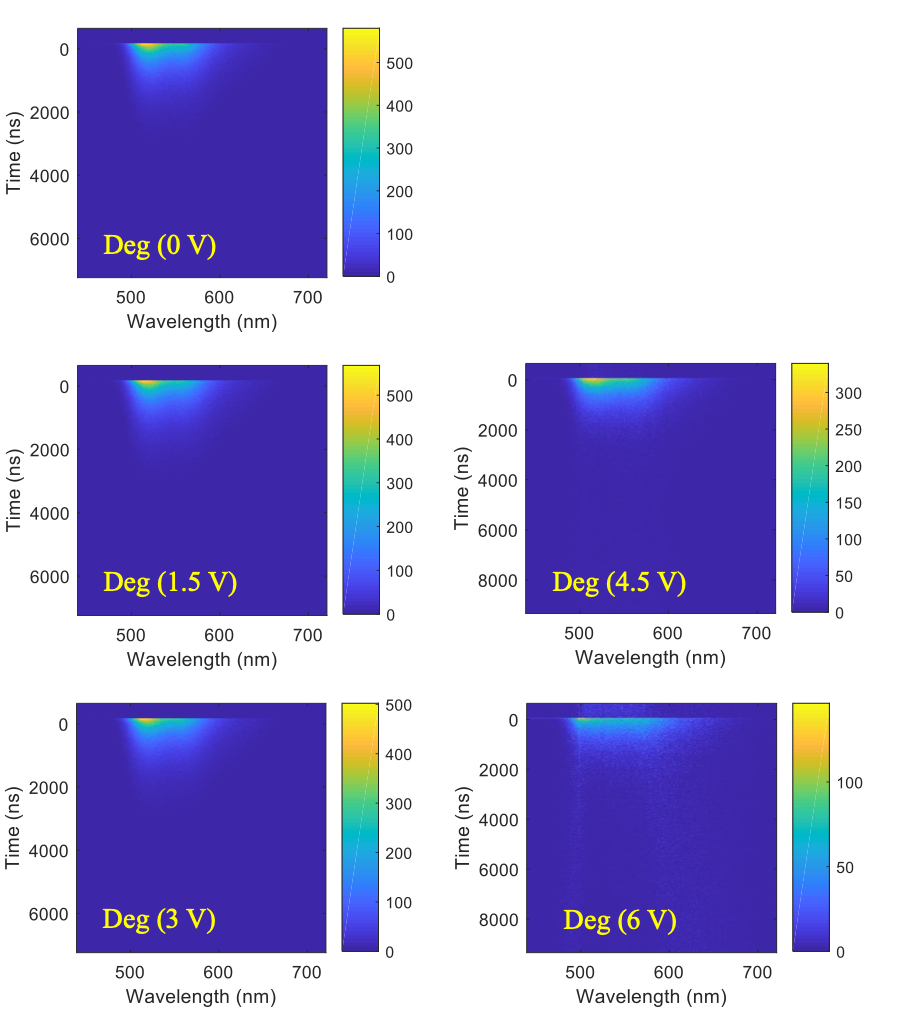
**

**Fig. S5: Raw EPS spectra (F_raw_) of the degraded device (Deg) under various external bias voltages.**

**Supplementary Fig. S5** contains the complete steady-state EPS spectra of the degraded OLED device under the same bias conditions as the reference. In contrast to the stable spectral features observed in the pristine device, the degraded device exhibits reduced intensity and narrower spectral width, particularly near the turn-on and high bias regions. These variations suggest a decline in exciton population and an increase in non-radiative emission, consistent with degradation effects.


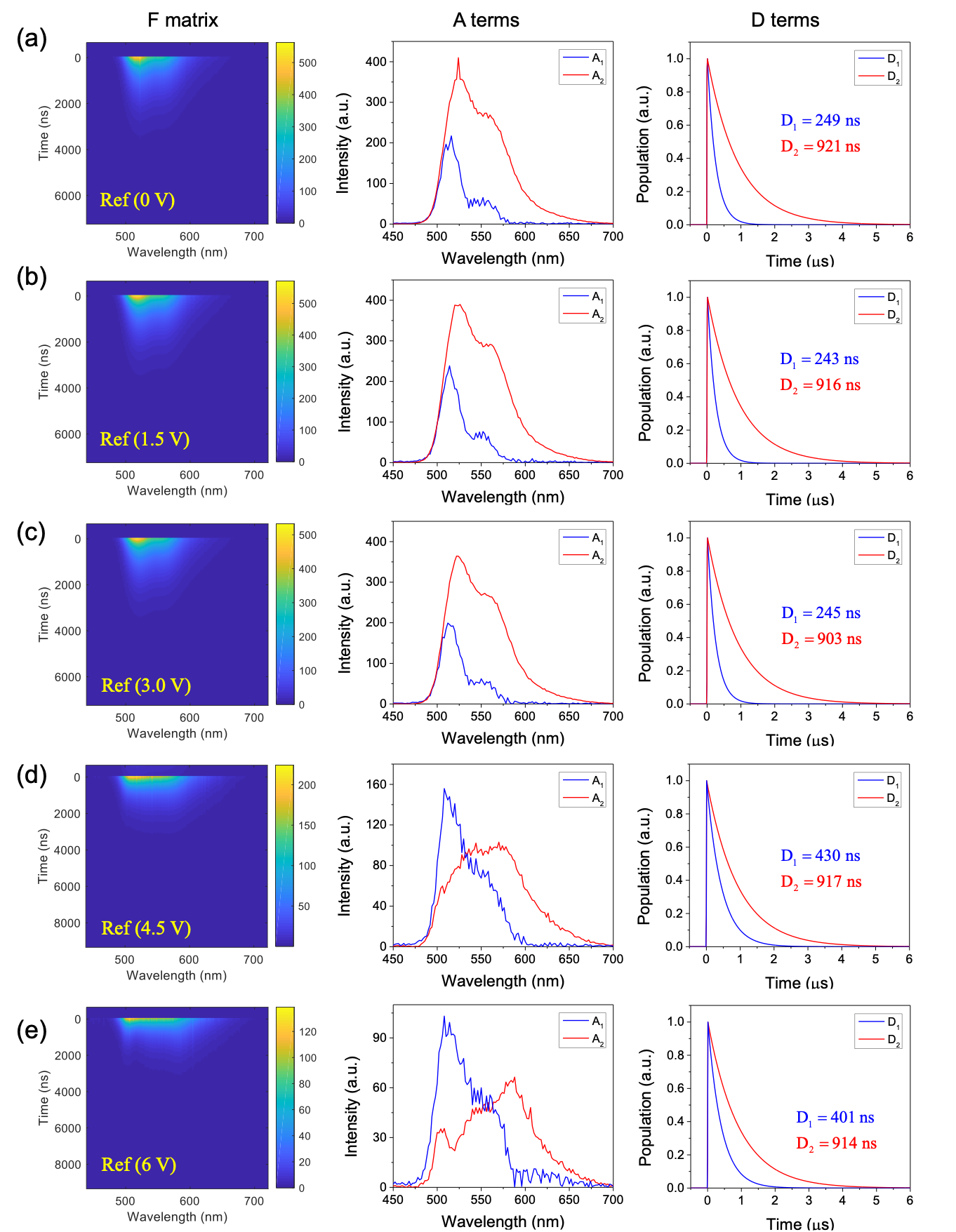


**Fig. S6: (a−e) Decay-associated spectra (DAS) analysis, showing F, A and D components of the EPS spectra for the reference device (Ref) under various external bias voltages (0−6V).**

A full decay-associated spectra (DAS) analysis of the reference device is provided in **Supplementary Fig. S6**. For each applied voltage, the spectral (**A**), temporal (**D**), and reconstructed (**F**) components are displayed. The **A** terms reveal distinct emission bands associated with target (**A**_1_) and parasitic (**A**_2_) excitons, while the **D** terms provide their corresponding lifetimes. The results confirm stable exciton dynamics across the operational voltage range.


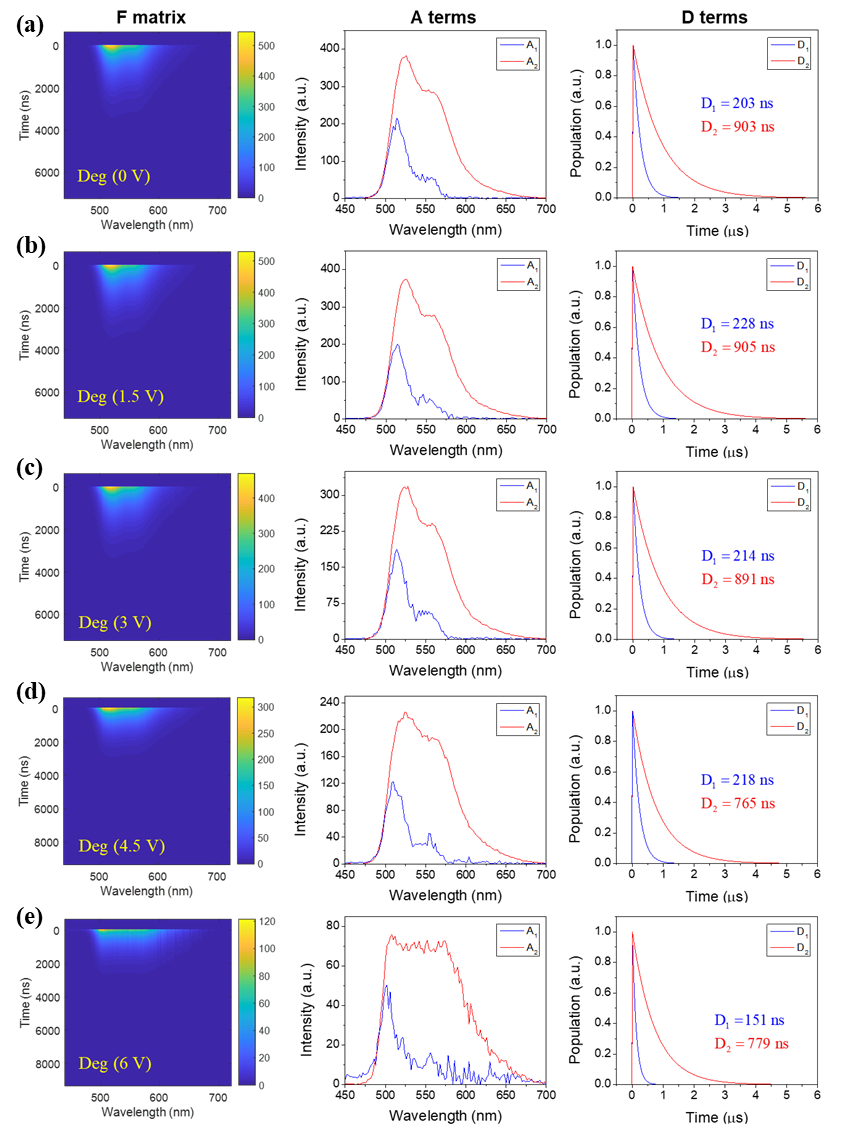


**Fig. S7: (a-e) Decay-associated spectra (DAS) analysis, showing F, A and D components of the EPS spectra for the degraded device (Deg) under various external bias voltages (0−6 V).**

**Supplementary Fig. S7** presents the complete DAS results for the degraded OLED device. Compared to the reference, above the turn-on voltage, the degraded device shows modified **A** terms and shortened **D** terms, indicating increased parasitic recombination and faster exciton decay. The reconstructed **F** terms also exhibit diminished intensity and spectral narrowing (see **Figure 4** in main text), which is consistent with the presence of trap-assisted quenching and interfacial degradation.

**
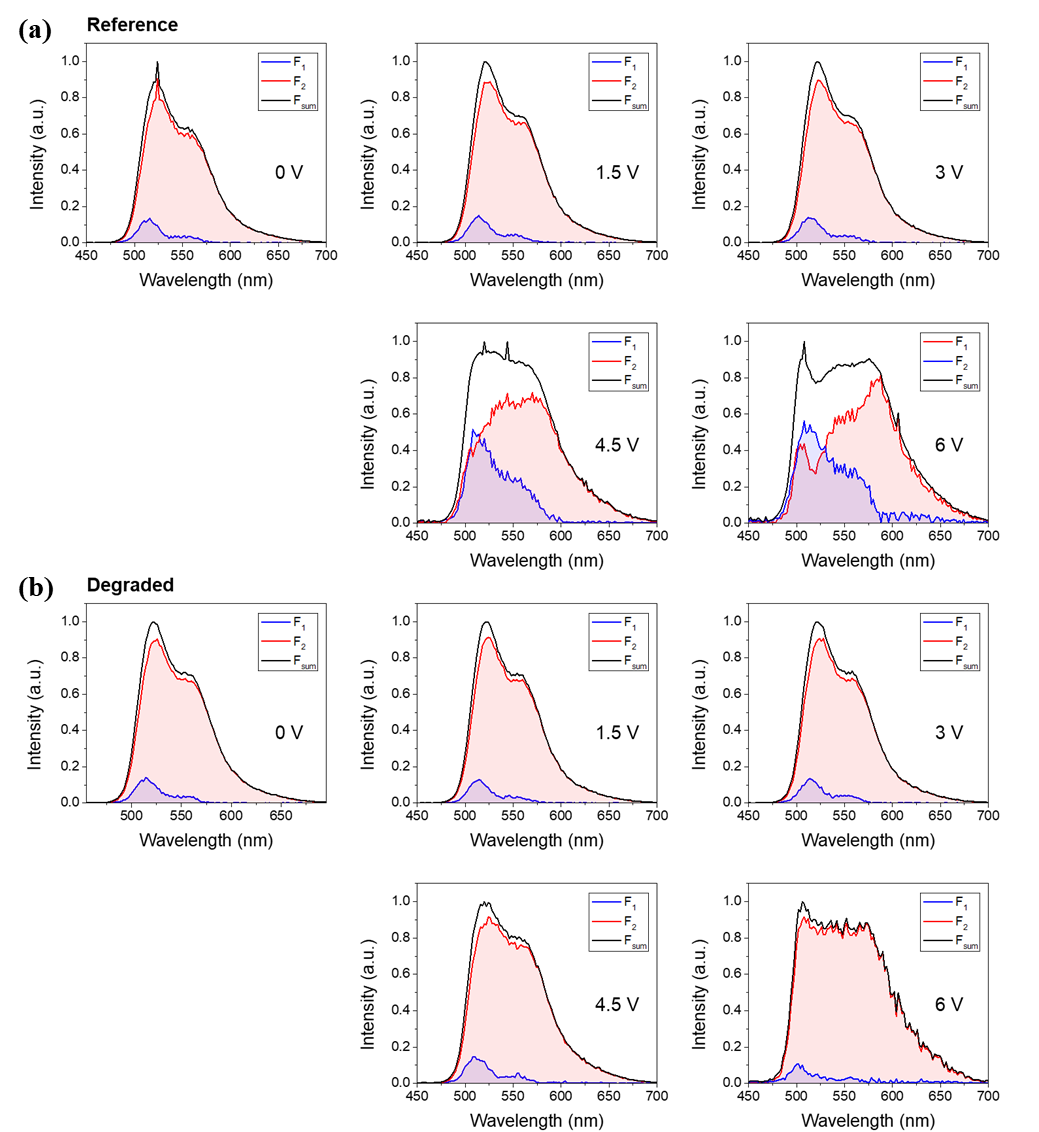
**

**Fig. S8: Deconvoluted ESP spectra, using the relation F=A×D, for (a) the reference device and (b) the degraded device at various bias voltages (0−6 V).**

**Supplementary Fig. S8** shows the mathematical deconvolution of the EPS spectra (**F** = **A**×**D**) used to extract the **F**_1_ and **F**_2_ terms for both devices. This procedure enables quantification of excitonic dynamics by resolving each spectral contribution into its temporal and spatial components.


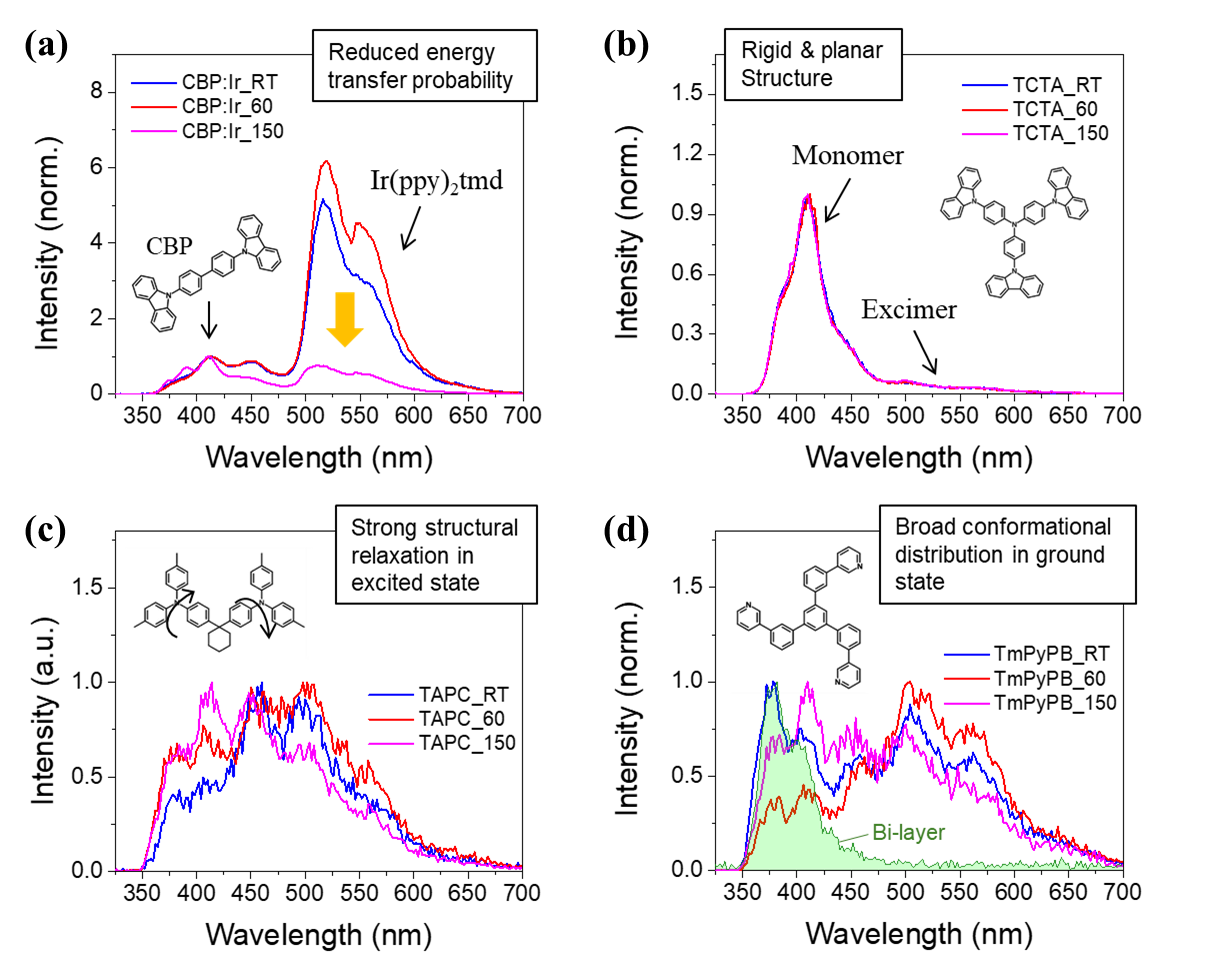


**Fig. S9: Steady-state PL spectra of reference (room temperature, RT) and thermally degraded (60 °C and 150 °C) single layers.** (a) EML (CBP:Ir), (b) TCTA, (c) TAPC, and (d) TmPyPB. For CBP:Ir, the PL spectra are normalized to the maximum PL intensity of the CBP matrix to highlight the reduced PL contribution from the dopant molecules, Ir(ppy)_2_tmd. For TmPyPB, the green-shaded band indicates the PL spectrum of the TmPyPB moiety in a bilayer film with the EML.

To identify the layer primarily affected by thermal degradation, each organic layer constituting the OLED device was fabricated as a single film on a Si substrate and subjected to three thermal stress conditions: **RT** (reference, room temperature), **60** (60 °C), and **150** (150 °C). The emission layer (CBP:Ir) exhibited dual emission bands at 420 nm and 525 nm, corresponding to CBP and Ir(ppy)_2_tmd, respectively. The dopant molecule’s PL band around 525 nm was dramatically quenched under the highest temperature condition (CBP:Ir**−**150), suggesting that degradation primarily perturbs the energy transfer process between CBP and the dopant in the EML.

TCTA exhibited negligible spectral change upon thermal stressing, consistent with its rigid, planar structure that limits non-radiative relaxations through skeletal motions. In contrast, TAPC and TmPyPB showed irregularly broadened and shifted emission bands, attributed to the broad conformational distribution of their flexible side groups that facilitates heterogeneous structural relaxation in the excited state.^5^ Therefore, one may expect that the excitonic dynamics of these flexible molecules are relatively insensitive to the thermal stress, due to the inherent conformational heterogeneity within the thin film.

Interestingly, the TmPyPB layer in the bilayer film (CBP:Ir/TmPyPB) exhibited a blue-shifted PL band, corresponding to the blue shoulder of the steady-state PL spectra observed in the single-layered films. This implies that interfacial interactions across the multilayers play a crucial role in constraining the conformational distribution during bilayer fabrication, thereby impacting the excitonic relaxation processes in the ETL.

**
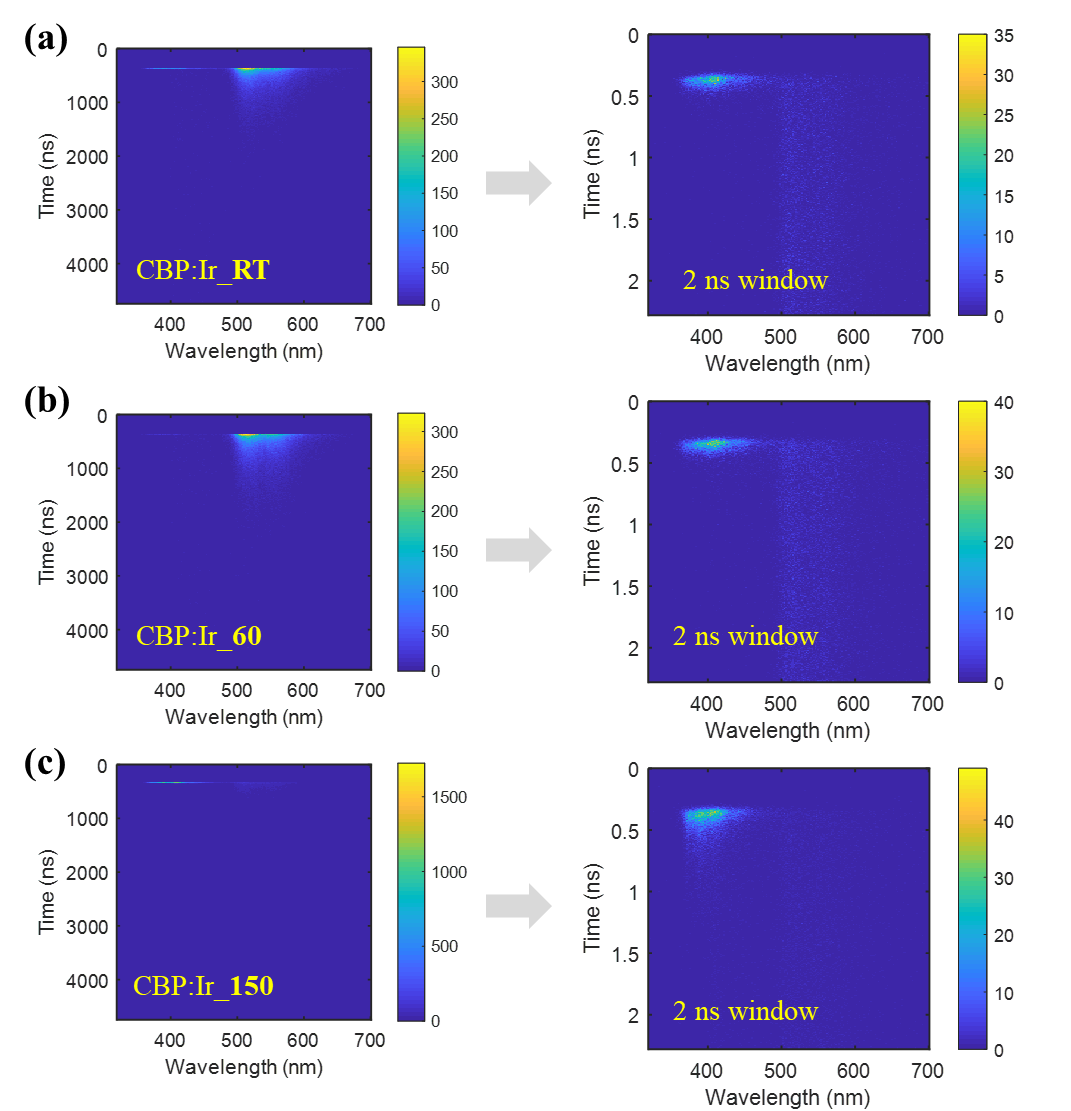
**

**Fig. S10: Raw TRPL spectra (wavelength vs. time) of CBP:Ir single-layer films under** (a) RT, (b) 60 °C, and (c) 150 °C thermal stress conditions. The left column shows a similar timescale to earlier plots (Fig. S5), while the right column shows enlarged plots with a time window of 2 ns.

To visualize the temporal evolution of excitonic emission in the emissive layer (EML), two-dimensional TRPL maps were obtained for CBP:Ir films. These maps reveal distinct decay behaviors for each spectral region. In the reference sample (**RT**), strong emission is observed from both the CBP host (~420 nm) and the Ir(ppy)_2_tmd dopant (~525 nm), with a clear temporal separation. Upon degradation at 60 °C and 150 °C, the dopant emission is gradually diminished, while the host emission becomes spectrally narrower. Especially in the 150 °C sample, the Ir emission is significantly quenched, and the CBP emission dominates with a faster decay. These trends confirm that thermal stress leads to degradation of the CBP matrix, affecting both energy transfer efficiency and exciton recombination dynamics within the EML.


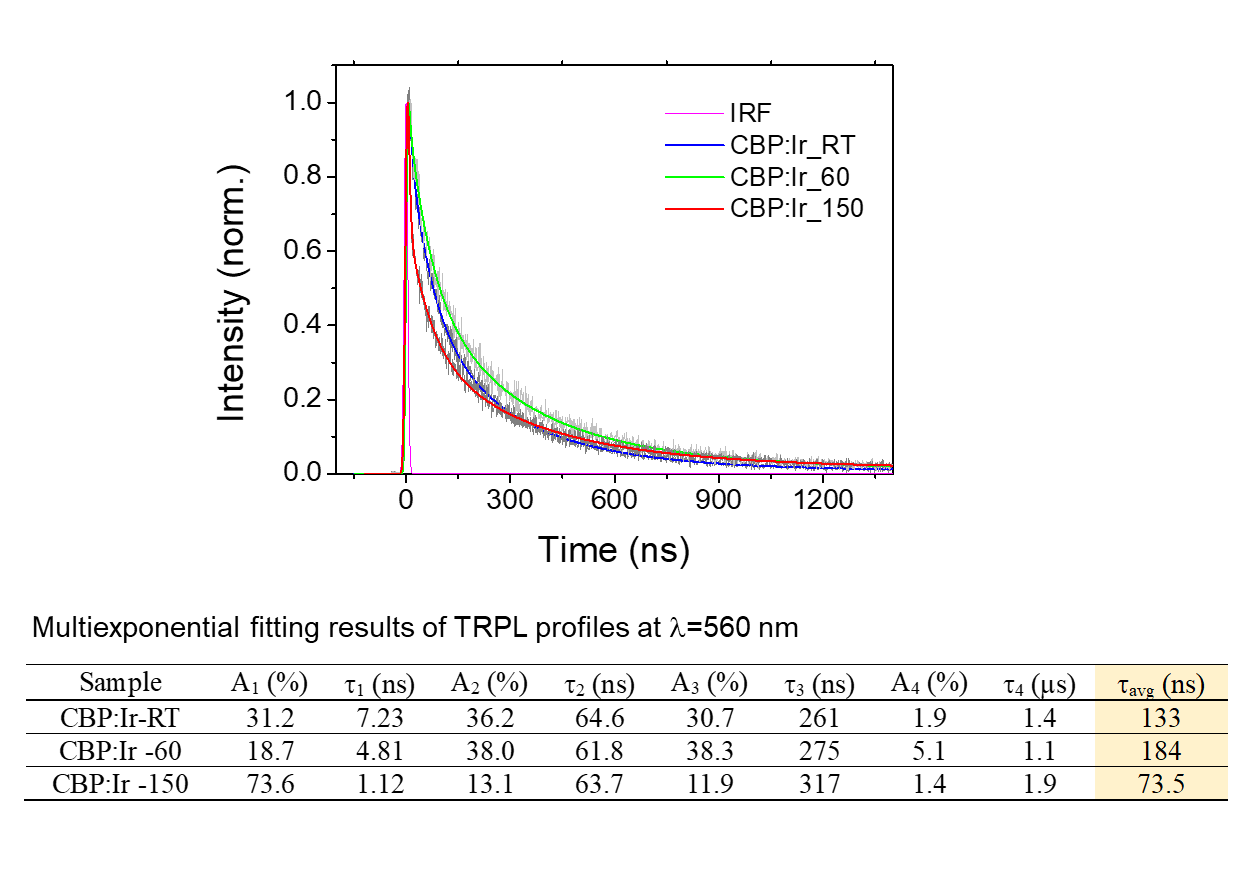


**Fig. S11: TRPL analysis of CBP:Ir single layers: PL time-profiles of the Ir dopant at 560 nm and their multi-exponential fitting results, for the three different thermally-stressed samples (RT, 60 °C, and 150 °C). IRF denotes the instrument response function.**


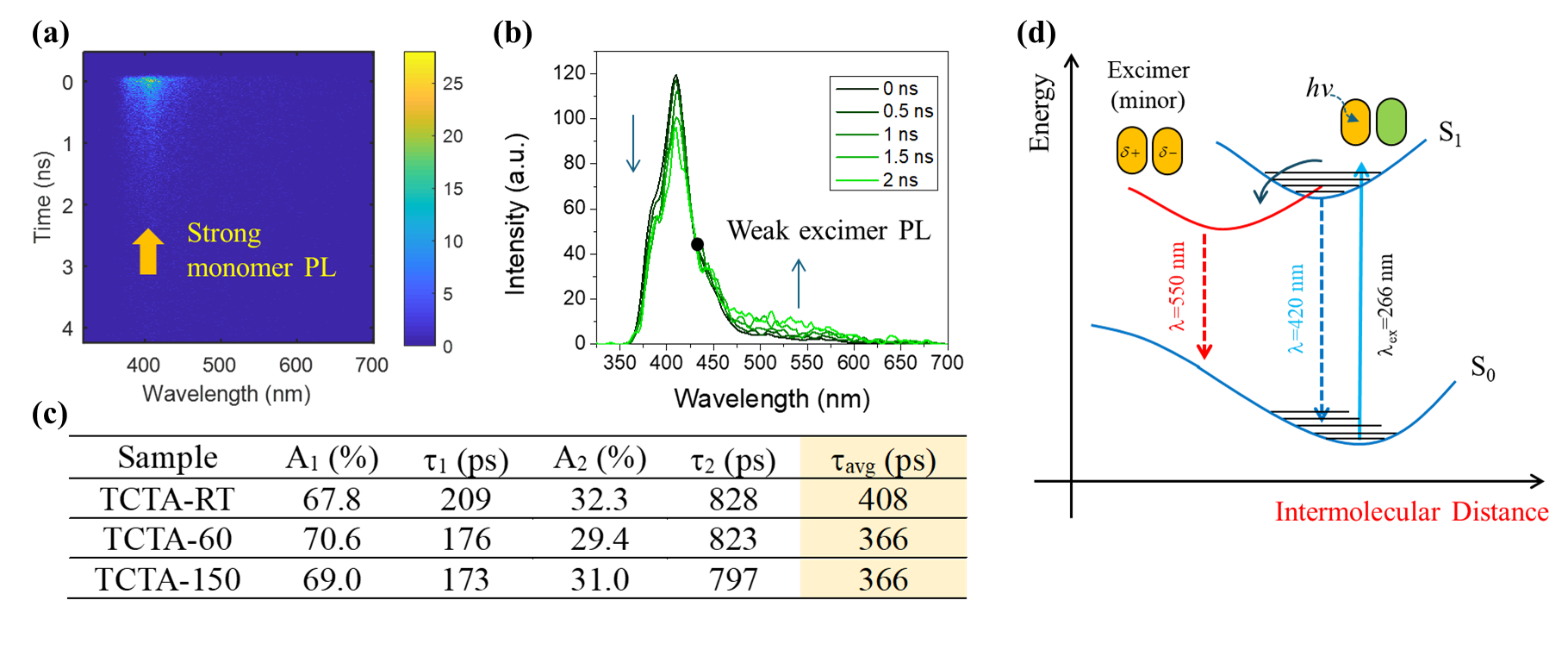


**Fig. S12: TRPL analysis of TCTA single layers:** (a) Picosecond TRPL spectrum of TCTA−RT in a 4 ns window, and (b) its time-resolved area-normalized emission spectrum (TRANES) at 5 different times, where the black dot indicates an iso-emissive point. (c) Multi-exponential fitting results for the spectrally integrated time-profiles of the three single-layer samples with different thermal stresses. (d) Schematic reaction diagram describing the weak excimer emission around 550 nm.

Picosecond TRPL analysis for the single-layered TCTA films confirmed that the weak PL band around 550 nm originates from the slow formation of excimers (**Fig. S12a-b**), suggesting that a small fraction of TCTA moieties form molecular aggregates with favorable intermolecular geometries for excimer formation. Notably, the average PL lifetime of the TCTA film decreased by only ~10 % even at the highest annealing temperature (**Fig. S12c**), confirming that the degraded EBL (TCTA) alone is not responsible for the distinct decline in device efficiency.


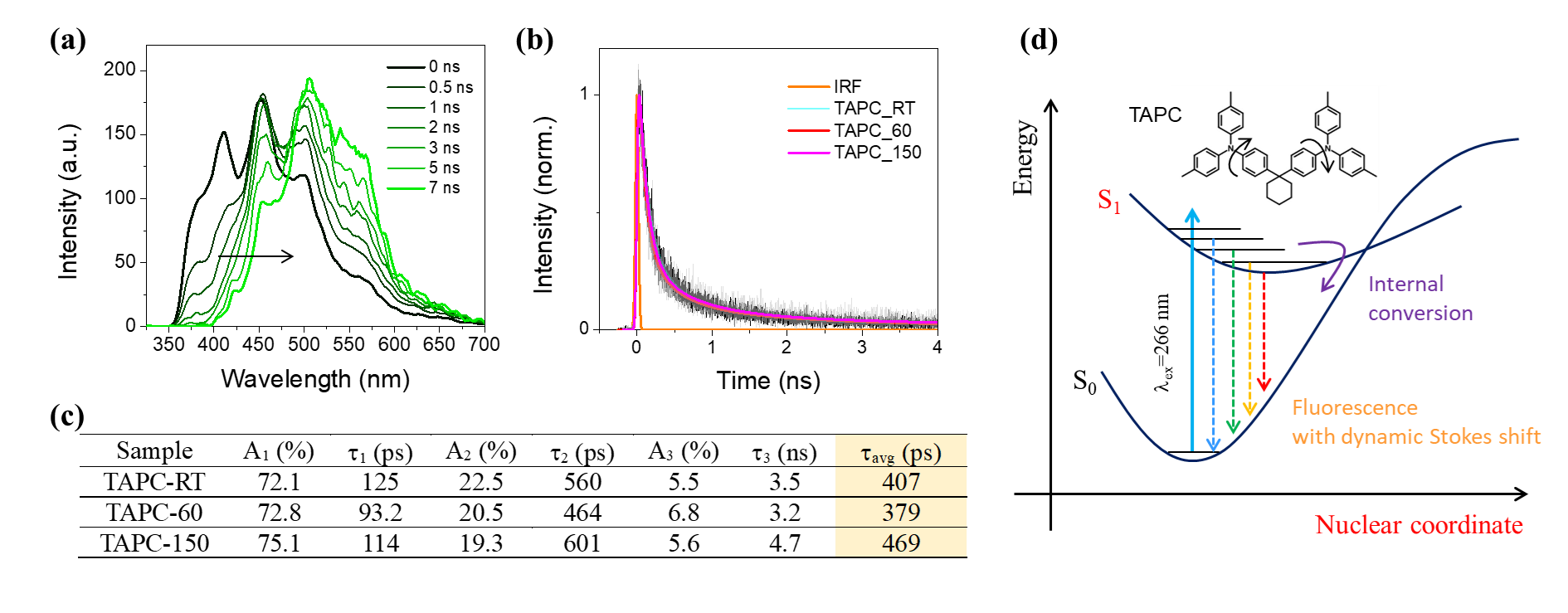


**Fig. S13: TRPL analysis of TAPC single layers:** (a) Time-resolved area-normalized emission spectra (TRANES) of TAPC−RT, (b) Spectrally integrated time-profiles of the three temperature-controlled samples, and (c) their multi-exponential fitting results. (d) Schematic energy diagram describing the dynamic Stokes shift via continuous structural relaxation in the excited state.

Single-layered TAPC films exhibited broad steady-state PL spectra with strong vibronic structures. As previously reported, the flexible side groups of TAPC induce characteristic structural relaxations in the excited state,^5^ resulting in a pronounced dynamic Stokes shift of the TRPL spectra (see **Fig. S13a**). Accordingly, the irregular spectral shifts of the PL spectra can be attributed to structural relaxations of TAPC embedded in the heterogeneous matrix environment of the degraded thin film. Nonetheless, the lifetimes of the spectrally integrated PL profiles remained similar under thermal degradation (**Fig. S13b-c**), indicating that the primary source of degradation in the OLED device is located in another layer.


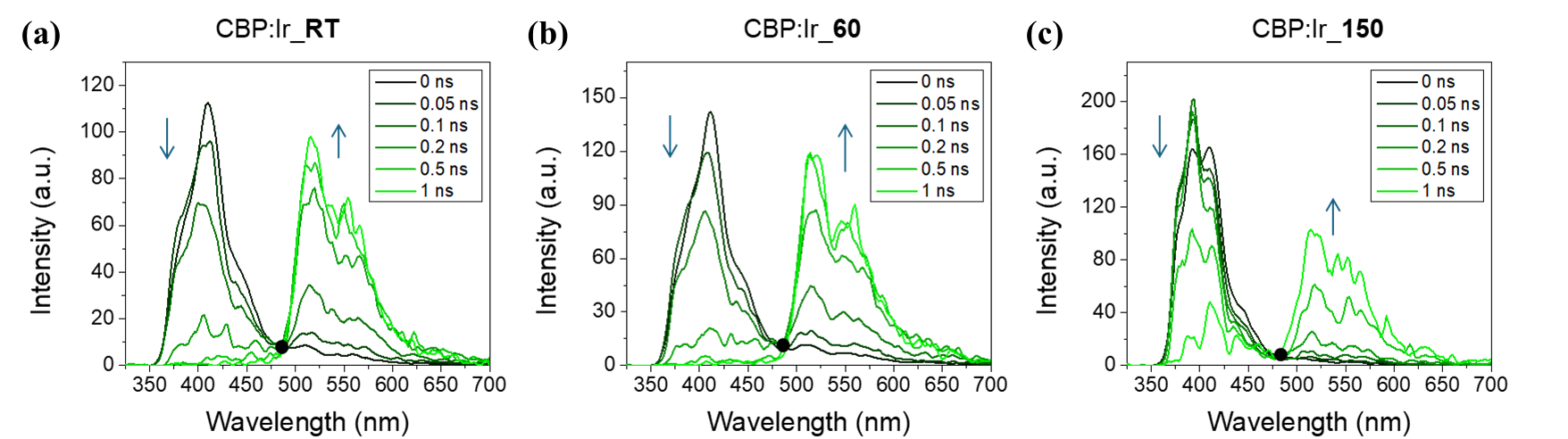


**Fig. S14: Time-resolved area-normalized emission spectra (TRANES) of CBP:Ir films under (a) RT, (b) 60 °C, and (c) 150 °C conditions.**

Comprehensive analysis for the picosecond TRPL spectra of the CBP:Ir samples revealed the role of the degraded CBP matrix as an effective excitonic scattering source. We conducted time-resolved area-normalized emission spectra (TRANES) analysis for the TRPL spectra as shown in **Fig. S14**, through which direct population transfer between two emissive states can be resolved by observing an iso-emissive point.^6^ All samples exhibited an iso-emissive point near 480 nm, indicating energy transfer from the host CBP (~420 nm) to the Ir(ppy)_2_tmd dopant (~525 nm). In the reference film (**RT,** **Fig. S14a**), the dopant emission progressively grows as the host emission decays, consistent with efficient host-to-dopant energy transfer. Upon moderate degradation at 60 °C **(Fig. S14b**), a similar trend is maintained, though with slightly reduced dopant intensity. However, the 150 °C-degraded film **(Fig. S14c**) shows a markedly reduced dopant signal and a narrower CBP emission, suggesting that excitons are increasingly scattered or quenched before successful transfer to the dopant. These findings support the hypothesis that thermal degradation of the CBP matrix introduces non-radiative pathways which interfere with the energy funneling process essential for high-efficiency OLED operation.


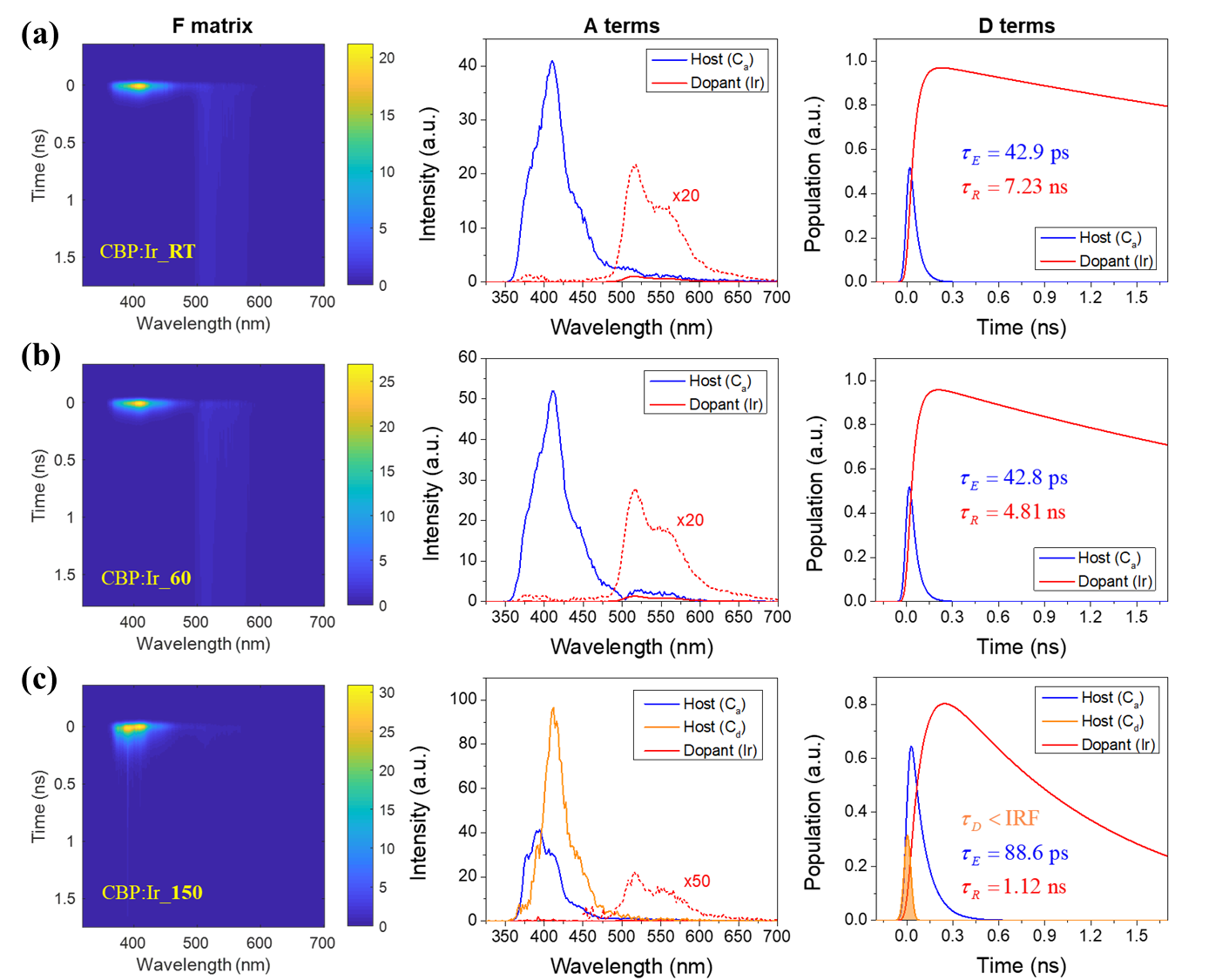


**Fig. S15: Species-associated spectra (SAS) analysis for the TRPL spectra of three thermally controlled single-layered CBP:Ir films.** For (a) CBP:Ir−RT and (b) CBP:Ir−60, a simple serial reaction scheme (C_a_🡪Ir🡪GS) was employed to confirm the energy transfer between the host and the dopant. For (c) CBP:Ir−150, an additional ultrafast scattering pathway (C_d_🡪GS) was included to identify the role of the degraded CBP matrix as a non-radiative scattering source.

To quantitatively model the exciton dynamics within the EML, TRPL spectra were analyzed using SAS with predefined kinetic reaction schemes (see main manuscript **Fig. 5**). For the reference (**RT**) and moderately degraded (**60** °C) films, a simple sequential model (**C_a_ → Ir → GS**) was sufficient, where **C**_a_ denotes the activated CBP host, **Ir** the dopant, and **GS** the ground state. Both conditions exhibited fast energy transfer (τ_E_ ≈ 43 ps) and relatively long dopant lifetimes (τ_R_ > 4 ns), indicating minimal disturbance in exciton migration and recombination. In contrast, for the highly degraded film (**150** °C), an additional decay pathway (**C_d_ → GS**) was required, corresponding to exciton quenching via damaged host sites (**C**_d_). The corresponding **A** spectrum for **C**_d_ displayed a sharp and intense component, while the associated **D** term was nearly instantaneous, reflecting ultrafast non-radiative decay (τ_D_ ≈ approximately IRF-limited). Furthermore, the host-to-dopant transfer was delayed (τ_E_ ≈ 89 ps), and dopant recombination was strongly suppressed, confirming that CBP degradation introduces scattering centers which hinder excitonic energy transfer and reduce radiative recombination efficiency.


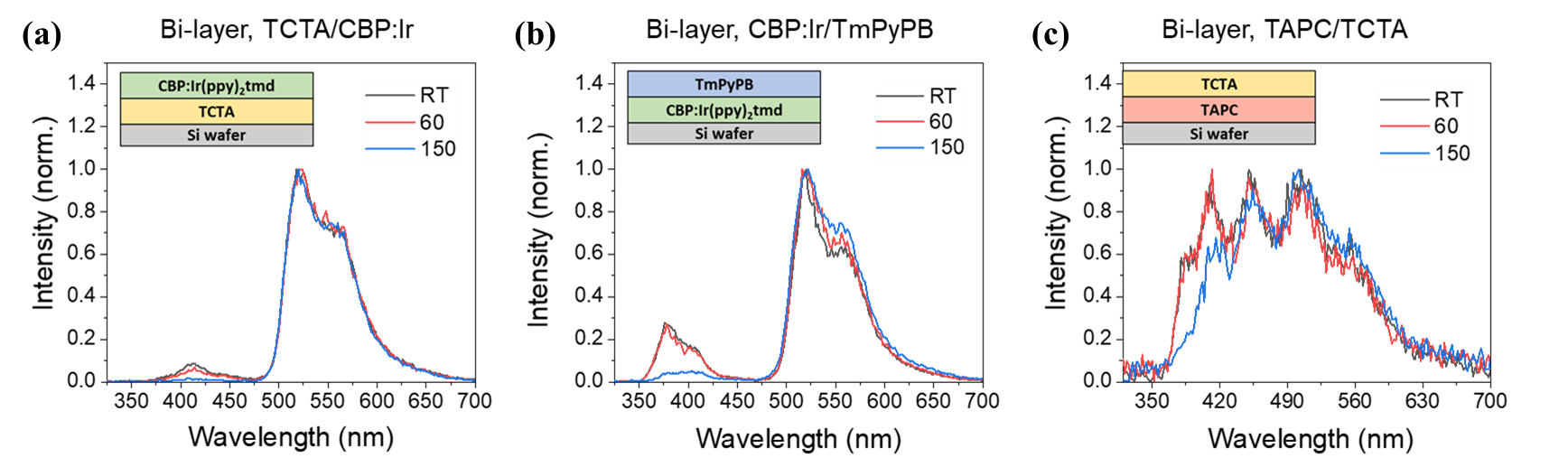


**Fig. S16: Steady-state PL spectra of bilayer films fabricated with actual device layer thicknesses:** (a) TCTA/CBP:Ir, (b) CBP:Ir/TmPyPB, and (c) TAPC/TCTA, under RT, 60 °C, and 150 °C conditions.

To replicate the real OLED architecture, bilayer films were fabricated using the same thickness parameters as the devices. Their steady-state PL spectra show highly convoluted emission features, with spectral components from both layers overlapping across the 350–550 nm range. In the TCTA/CBP:Ir (a) and CBP:Ir/TmPyPB (b) bilayers, dopant emission around 525 nm was more intense than in the single-layer EML, suggesting that interfacial interactions reduce non-radiative losses at the exposed EML surface. Conversely, the TAPC/TCTA (c) bilayer exhibited no significant spectral enhancement, indicating negligible excitonic coupling between these HTL components. This behavior supports the hypothesis that interfacial configurations involving the EML can modify exciton relaxation dynamics, especially when adjacent layers actively participate in exciton confinement or transfer processes.


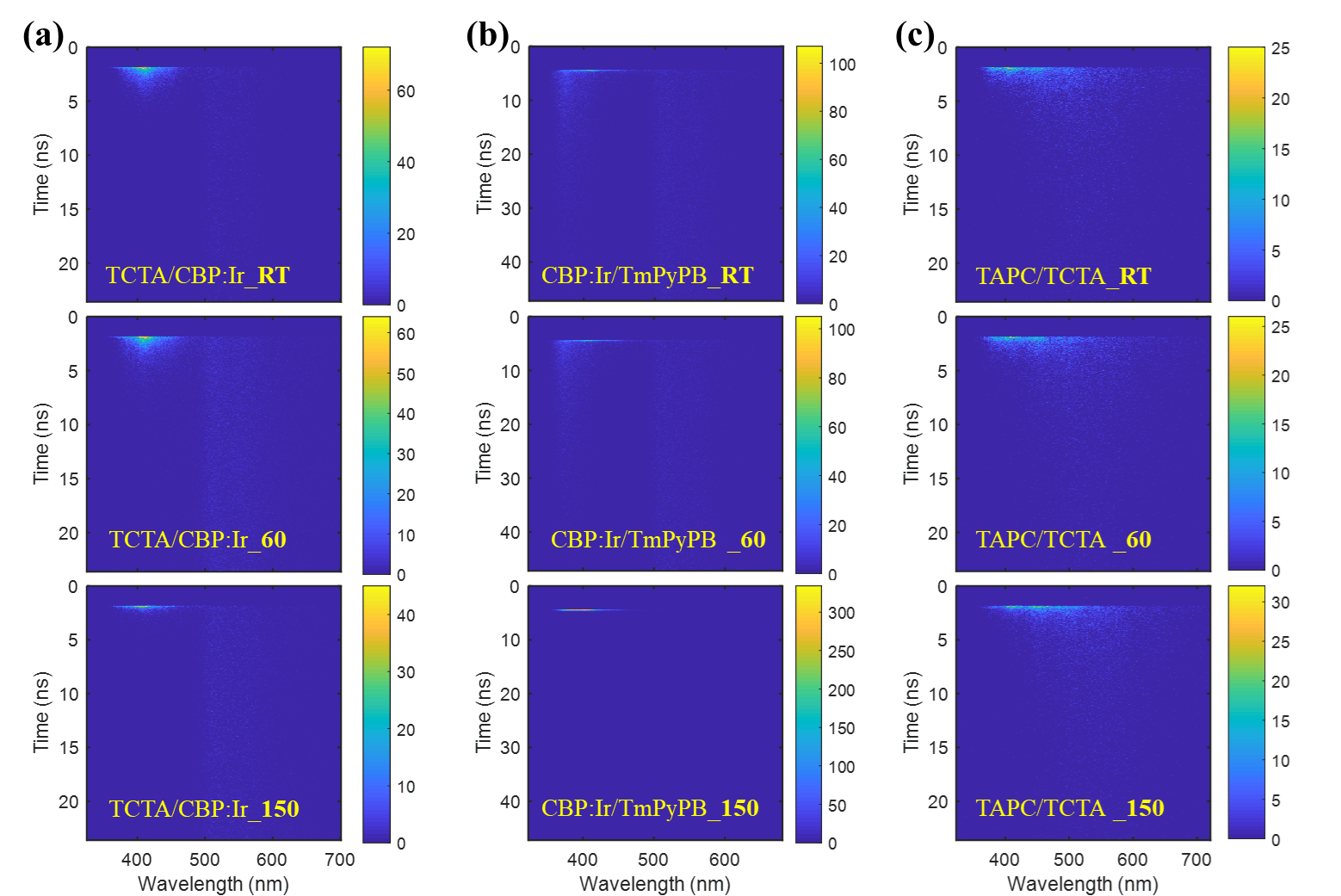


**Fig. S17: Raw TRPL maps of bilayer films:** (a) TCTA/CBP:Ir, (b) CBP:Ir/TmPyPB, and (c) TAPC/TCTA, under RT, 60 °C, and 150 °C conditions.

Raw TRPL maps (**Fig. S17**) were collected for the bilayer films to investigate their exciton dynamics with high temporal and spectral resolution. In the TCTA/CBP:Ir bilayer (**a**), spectral evolution and delayed emission features near 480–525 nm are observed, suggesting additional interfacial energy transfer from the TCTA layer to the dopant in the EML. The CBP:Ir/TmPyPB bilayer (**b**) exhibited a strong and persistent emission band near 380 nm originating from TmPyPB, which was gradually quenched with increased thermal stress. In contrast, the TAPC/TCTA bilayer (**c**) displayed broad and structured emission features without noticeable changes in temporal evolution, consistent with minimal interfacial excitonic interactions. These raw TRPL maps support the analysis presented in the main manuscript, confirming that only specific bilayer configurations such as TCTA/CBP:Ir exhibit substantial interfacial exciton transfer processes.


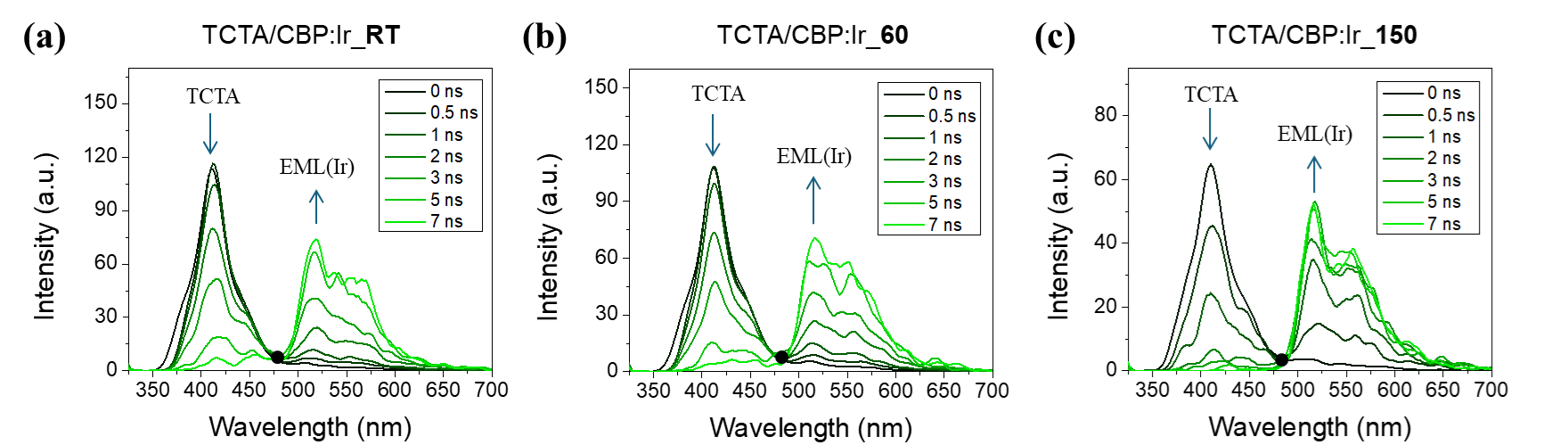


**Fig. S18: TRANES of TCTA/CBP:Ir bilayer films under (a) RT, (b) 60 °C, and (c) 150 °C conditions.**

TRANES analysis was performed on the three TCTA/CBP:Ir bilayer films to investigate interfacial excitonic energy transfer **(Fig. S18)**. All samples exhibited a distinct iso-emissive point around 480 nm, indicating a population transfer from TCTA (donor) to the Ir dopant (acceptor) in CBP:Ir. It is important to note that the PL band around 410 nm originates primarily from the TCTA layer, not from the CBP, as all the films displayed similar spectral features in shape along with slow population decay (> 0.5 ns). This behavior is in sharp contrast to that of the CBP:Ir single-layer films (**Fig. S14**), where the CBP host matrix exhibited fast population decay (τ_E_≈40 ps) within a 1 ns time-window, and the spectral shape of the CBP emission under the highest degradation condition was completely different to those of the other temperature-controlled single-layered films. Therefore, for the bilayer films, the iso-emissive points resolved in the TRANES analysis beyond 0.5 ns time-delay can be solely attributed to direct interfacial energy transfer between the TCTA layer and the dopant in the EML. This result validates the hypothesis that excitonic interactions at the TCTA/CBP:Ir junction play a critical role in multilayer OLED degradation behavior.


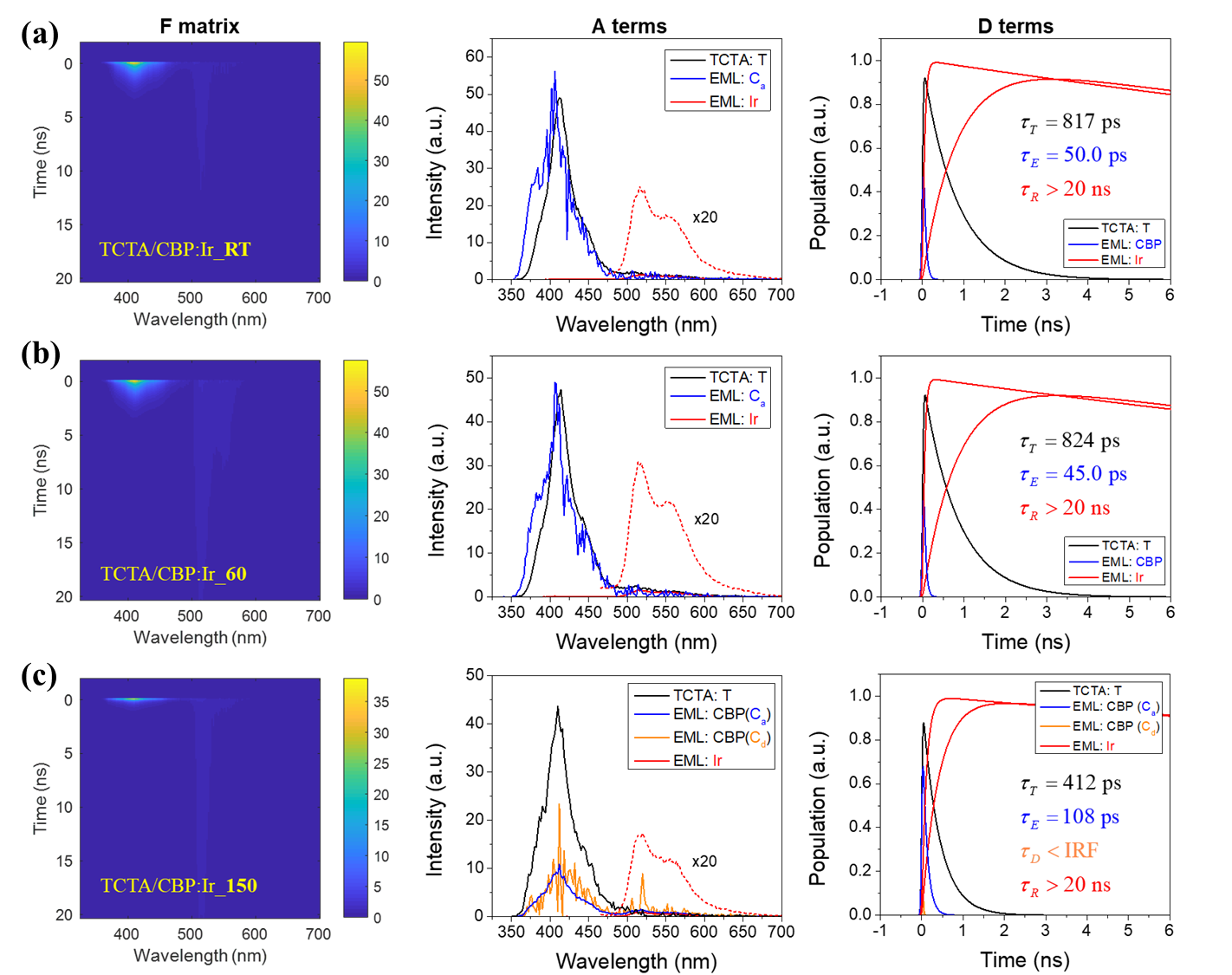


**Fig. S19: Species-associated spectra (SAS) analysis for the TRPL spectra of three TCTA/CBP:Ir bilayers under (a) reference (RT) and (b) 60°C, (c) 150°C degradation conditions.** For the TCTA/CBP:Ir−150, the ultrafast exciton scattering pathway associated with the damaged CBP matrix (**C_d_**) was included in the fitting model. In the reaction schemes, T denotes TCTA.

Based on the TRANES results, the TRPL spectra were further analyzed using the SAS procedure with predefined kinetic reaction schemes incorporating interfacial energy transfer pathways (**T → Ir → GS**), as illustrated in main manuscript **Fig. 5** (upper panel). The reaction model also included excitonic relaxation routes previously identified in the single-layer films (**C_a_ → Ir → GS and C_d_ → GS**) to ensure consistency across sample types. For the CBP:Ir/TCTA−**RT** and CBP:Ir/TCTA−**60** samples, SAS successfully resolved two closely overlapping PL bands corresponding to TCTA (λ_max_ ≈ 410 nm) and CBP (λ_max_ ≈ 400 nm). The associated **D** terms confirmed the presence of slow interfacial exciton transfer (τ_T_ ≈ 820 ps) from TCTA to the dopant Ir, along with relatively fast host-to-dopant energy transfer within the EML (τ_E_ ≈ 45–50 ps), consistent with an intact multilayer environment.

In the highly degraded TCTA/CBP:Ir−**150** sample, the spectral features became more complex due to the emergence of another exciton scattering source (**C**_d_), corresponding to damaged CBP sites. Although the spectral intensity of this quenching channel (**A** term) was diminished—likely due to convolution with the IRF (~50 ps)—ultrafast decay (τ_D_ approximately IRF-limited) was clearly observed. Notably, the τ_T_ value decreased to ~410 ps, suggesting enhanced interfacial excitonic coupling under degradation. This indicates that degradation of the EBL may locally increase the electronic coupling across the EML/EBL interface, accelerating energy transfer toward the dopant in the presence of interfacial disorder, thereby contributing to the increased parasitic exciton generation observed in degraded devices.


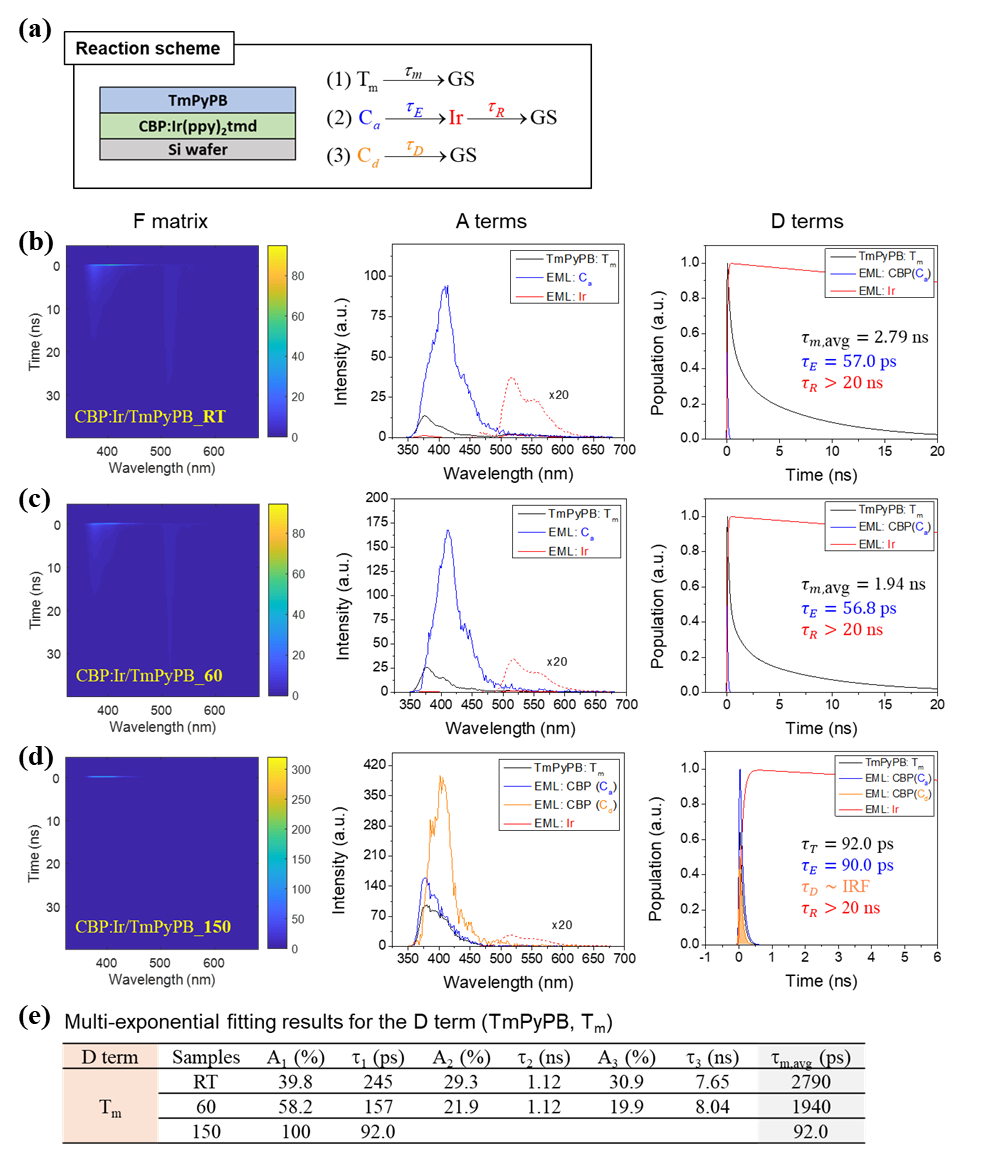


**Fig. S20: Species-associated spectra (SAS) analysis of the TRPL spectra from the reaction scheme in (a) for three CBP:Ir/TmPyPB bilayers under (b) reference and (c, d) degradation conditions (60 °C, 150 °C). (e) Multi-exponential fitting results for the D term (TmPyPB).** For CBP:Ir/TmPyPB−150, the ultrafast exciton scattering pathway associated with the damaged CBP matrix (**C_d_**) was included in the fitting model. In the reaction schemes, **T_m_** denotes TmPyPB.

The CBP:Ir/TmPyPB bilayer films exhibited similar exciton kinetics, except for the interfacial energy transfer. Under reference conditions (**RT**), the TRPL spectra were fitted using a parallel reaction model that includes independent excitonic relaxation in the TmPyPB layer (**T_m_ → GS**), along with host-to-dopant energy transfer (**C_a_ → Ir → GS**) and a degradation-induced ultrafast quenching channel (**C_d_ → GS**) for the CBP matrix. A long PL decay exceeding 1 ns around 380 nm can be attributed to the PL of the TmPyPB layer (**T_m_**🡪**GS**), since the characteristic energy transfer (**C_a_**🡪**Ir**🡪**GS**) and ultrafast quenching (**C_d_**🡪**GS**) processes resolved in the bilayer films were almost identical to those observed in the single-layer films (**Fig. S15**). These observations allow for including excitonic dynamics of TmPyPB as the additional independent relaxation pathway, implying negligible interfacial excitonic coupling between the EML and TmPyPB layer.

The SAS results show a distinct, blue-shifted PL band centered around 380 nm originating from the TmPyPB layer, which exhibited prolonged average lifetime (τ_m,avg_) due to its structurally confined conformations in the bilayer environment. However, upon thermal degradation, this TmPyPB emission was strongly quenched, with a substantial reduction in its average PL lifetime (τ_avg_) from 2.79 ns to 92 ps. The **D** term extracted for TmPyPB clearly shows this degradation trend (**Fig. S20e**). These results indicate that while CBP:Ir and TmPyPB layers show limited interfacial excitonic coupling, the degradation of the ETL (TmPyPB) critically impacts exciton migration efficiency toward the EML through enhanced non-radiative vibronic relaxation.


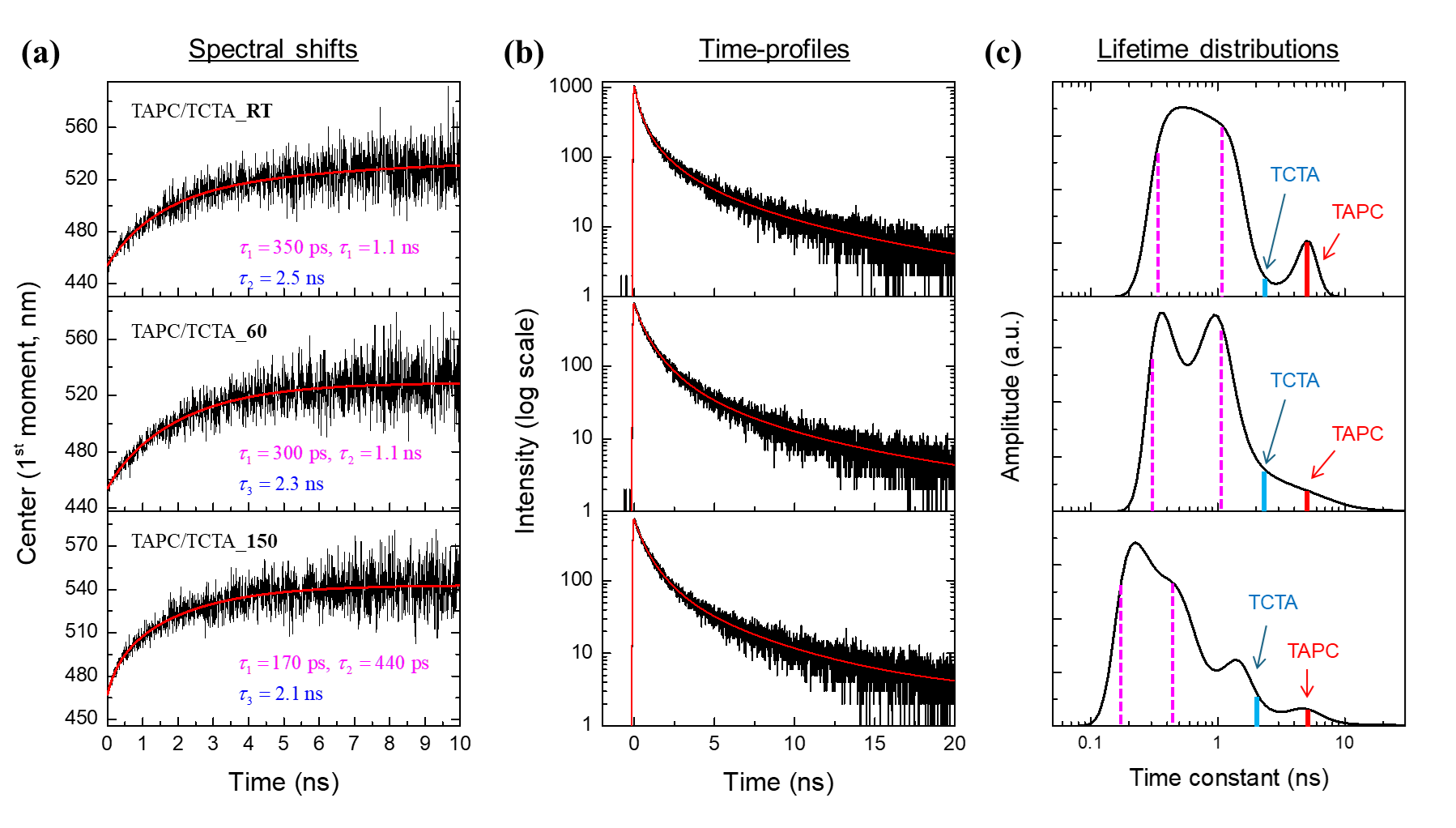


**Fig. S21:** **Photophysical analysis of the TAPC/TCTA bilayer films under different degradation conditions (RT, 60 °C, 150 °C):** (a) Spectral shifts with intensity-weighted center wavelength (1^st^ moment) shown in red; (b) spectrally integrated time profiles of the TRPL spectra. (c) PL lifetime distributions obtained by the maximum entropy method (MEM). The two dashed lines (pink) and one solid line (blue) correspond to the time-constants retrieved from the multi-exponential fitting of the 1^st^ moment traces.

Unfortunately, SAS analysis could not be applied to examine the exciton dynamics of the TCTA/TAPC bilayer films, as it assumes the **A** spectra are time-invariant in shape. Unlike the case of the EML/TmPyPB structure, the strong and broad vibronic progression in the PL spectra of TAPC persisted in the bilayer films (**Figs. S16 and S17c**), indicating that TAPC undergoes continuous structural relaxation in the excited state within the multilayer structure. In this context, we examined the TRPL spectra by comparing the timescales of the dynamic Stokes shift with corresponding lifetime distributions of the population relaxations, as shown in **Fig. S21c**.

The timescales of the dynamics Stokes shifts observed in the TRPL maps (**Fig. S17c**) were estimated from the intensity-weighted spectral center shifts (1^st^ moments) (see panel (a)). Both the intrinsic structural relaxations (τ_1_ and τ_2_) of TAPC and the population decay (τ_3_) of the rigid TCTA contribute to the dynamic redshifts of the spectra toward 520~540 nm. Notably, the PL spectra of TCTA around 410 nm remain invariant in both single-layer and bilayer films (see **Fig. S9b and S18**), indicating that population decay of TCTA also contributes to the observed redshift in the TRPL spectra. As shown in **Fig. S21b-c**, PL lifetime distributions were retrieved using the maximum entropy method (MEM).^7^ In these distributions, the three shifting components identified in the 1^st^ moment analysis are indicated by two dashed (pink) and one solid (blue) vertical lines. Based on this comparison, we confidently assign the time-constant band around 5 ns (red solid lines) to the population relaxation of fully relaxed TAPC, as the 1^st^ moment trace does not exhibit a corresponding long-timescale shift near the spectral center of 520~540 nm. Importantly, the exciton recombination rate (~ 5 ns) remained unchanged despite thermal degradation, confirming that the primary degradation effects originate from other layers, such as the EML and ETL.

Qualitatively, the two lifetime bands below 1.5 ns closely match the timescales of spectral shifts (pink vertical lines), implying that most TAPC molecules in the HTL undergo rapid structural relaxation in the excited state. These relaxations may facilitate population relaxation to the ground state or modulate the magnitude of transition dipole moment (non-Condon effect). As thermal stress increases, these structural relaxations become further accelerated, implying that the degraded HTL matrix permits faster skeletal motions of TAPC side groups due to a less confined environment. The assignment of the PL lifetime of TCTA in the bilayer films remains unclear. However, the third band around 1.5−2 ns (blue vertical line) may be associated with excitonic recombination within the TCTA layer. Given that the TAPC layer is about 3.5 times thicker than the TCTA layer, the PL intensity of the HTL around 410 nm contributes only marginally to the overall PL spectrum. Consequently, the dynamic Stokes shifts of the bilayer emissions are expected to be dominated by the HTL, leading to strong and broad lifetime bands below 1.5 ns in the MEM analysis. In this context, we argue that the weak lifetime band around 2 ns is attributed to the prolonged PL lifetime of TCTA in the bilayer films.


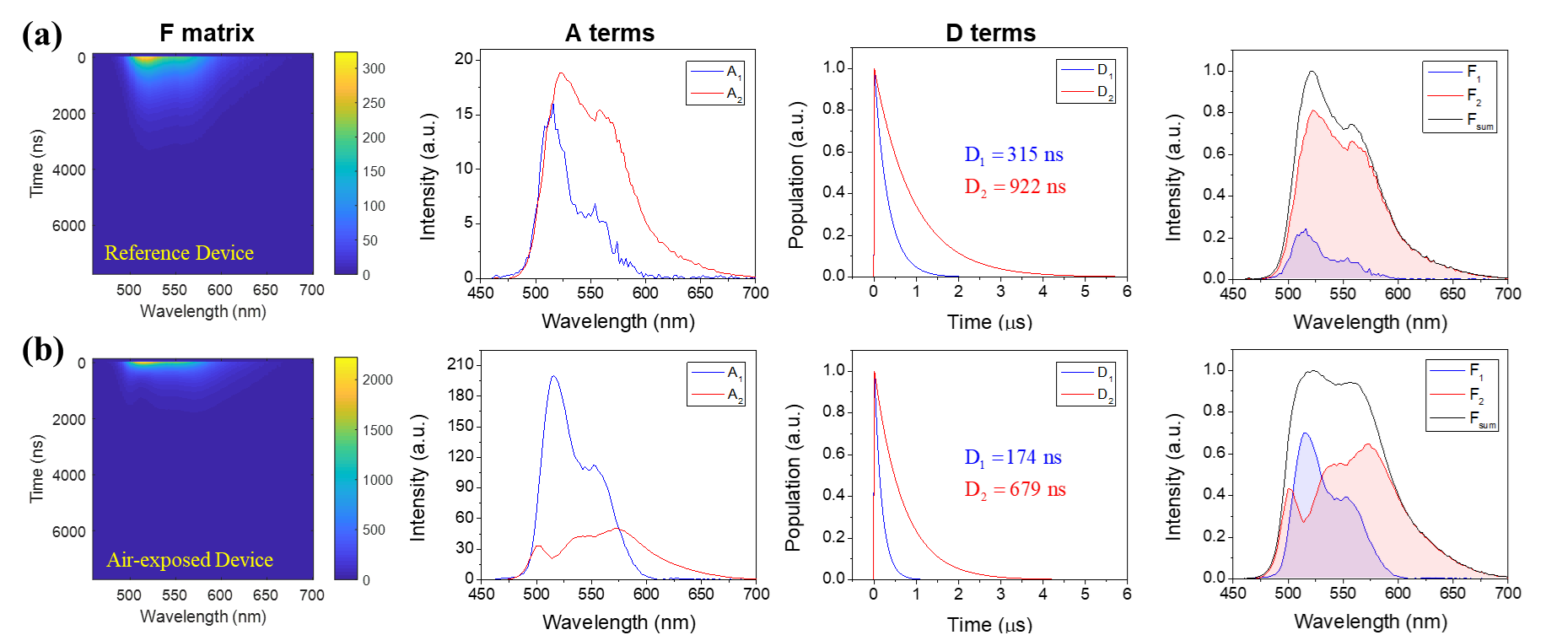


**Fig. S22:** **Decay-associated spectra (DAS) analysis of (a) the reference device and (b) the air-exposed OLED structure incorporating a spacer layer between the EML and EBL.** The A_2_ component, associated with parasitic excitons at the EML/EBL interface, was significantly suppressed compared to the reference device.

To further confirm the role of the EML/EBL interface in exciton scattering, air-exposed OLED structure was designed by introducing a thin non-emissive spacer layer between the EML and EBL to physically decouple the interfacial region. As revealed by DAS analysis (**Fig. S22**), the A_2_ component—previously identified as a spectral signature of interfacial parasitic excitons—was markedly reduced in the air-exposed device. This suppression indicates that direct contact between the doped EML and TCTA is essential for the formation of parasite excitons under thermal stress. The observed spectral simplification in the air-exposed configuration strongly supports the hypothesis that degradation-induced exciton scattering primarily originates at the EML/EBL interface.

**Reference**

1 Deng, Y. *et al.* Deciphering exciton-generation processes in quantum-dot electroluminescence. *Nature communications* **11**, 2309 (2020).

2 Kang, J. *et al.* Time‐Resolved Electroluminescence Study for the Effect of Charge Traps on the Luminescence Properties of Organic Light‐Emitting Diodes. *physica status solidi (a)* **217**, 2000081 (2020).

3 Cheng, Y. *et al.* Electronic structural insight into high‐performance quantum dot light‐emitting diodes. *Advanced Functional Materials* **32**, 2207974 (2022).

4 Lee, C. M. *et al.* Operando analysis for understanding the parasite exciton dynamics in blue phosphorescent OLEDs using electrically pumped spectroscopy. *Cell Reports Physical Science* **5** (2024).

5 Ha, I. *et al.* Exploring the Origin of White Emission in TAPC: Electron Transport Layer Based Exciplex Devices. *Advanced Optical Materials* **13**, 2403378 (2025).

6 Koti, A., Krishna, M. & Periasamy, N. Time-resolved area-normalized emission spectroscopy (TRANES): a novel method for confirming emission from two excited states. *The Journal of Physical Chemistry A* **105**, 1767-1771 (2001).

7 Kumar, A. T., Zhu, L., Christian, J., Demidov, A. A. & Champion, P. M. On the rate distribution analysis of kinetic data using the maximum entropy method: Applications to myoglobin relaxation on the nanosecond and femtosecond timescales. *The Journal of Physical Chemistry B* **105**, 7847-7856 (2001).
